# Differential genetic diagnoses of adult post-lingual hearing loss according to the audiogram pattern and novel candidate gene evaluation

**DOI:** 10.1007/s00439-021-02367-z

**Published:** 2021-09-14

**Authors:** John Hoon Rim, Byunghwa Noh, Young Ik Koh, Sun Young Joo, Kyung Seok Oh, Kyumin Kim, Jung Ah Kim, Da Hye Kim, Hye-Youn Kim, Jee Eun Yoo, Seung-Tae Lee, Jin Woong Bok, Min Goo Lee, Jinsei Jung, Jae Young Choi, Heon Yung Gee

**Affiliations:** 1 Department of Pharmacology, Graduate School of Medical Science, Brain Korea 21 Project, Yonsei University College of Medicine, 50-1 Yonsei-ro, Seodaemun-gu, Seoul, 03722 Republic of Korea; 2 Department of Medicine, Physician-Scientist Program, Yonsei University Graduate School of Medicine, Seoul, 03722 Republic of Korea; 3 Department of Laboratory Medicine, Yonsei University College of Medicine, Seoul, 03722 Republic of Korea; 4 Department of Otorhinolaryngology, Graduate School of Medical Science, Brain Korea 21 Project, Yonsei University College of Medicine, 50-1 Yonsei-ro, Seodaemun-gu, Seoul, 03722 Republic of Korea; 5 Graduate School of Medical Science, Brain Korea 21 Project, Yonsei University College of Medicine, Seoul, 03722 Republic of Korea; 6 Department of Anatomy, Yonsei University College of Medicine, Seoul, 03722 Korea

## Abstract

Ski-slope hearing loss (HL), which refers to increased auditory threshold at high frequencies, is common in adults. However, genetic contributions to this post-lingual HL remain largely unknown. Here, we prospectively investigated deafness-associated and novel candidate genes causing ski-slope HL. We analyzed 192 families with post-lingual HL via gene panel and/or exome sequencing. With an overall molecular diagnostic rate of 35.4% (68/192) in post-lingual HL, ski-slope HL showed a lower diagnostic rate (30.7%) compared with other conditions (40.7%). In patients who showed HL onset before the age of 40, genetic diagnostic probability was significantly lower for ski-slope HL than for other conditions. Further analysis of 51 genetically undiagnosed patients in the ski-slope HL group identified three variants in delta-like ligand 1 (*DLL1*), a Notch ligand, which presented in vitro gain-of-function effects on Notch downstream signaling. In conclusion, genetic diagnostic rates in post-lingual HL varied according to audiogram patterns with age-of-onset as a confounding factor. *DLL1* was identified as a candidate gene causing ski-slope HL.

**Supplementary Information:**

The online version contains supplementary material available at 10.1007/s00439-021-02367-z.

## Introduction

Hearing loss (HL) is the most common sensory disorder, affecting approximately one in every 500 newborns worldwide (Wilson et al. 2017). Since 2016, the application of next-generation sequencing (NGS) technology to determine the genetic causes of non-syndromic HL has enabled the identification of 26 additional genes for which variants have been linked to non-syndromic HL development; currently, more than 100 genes are implicated in the disease. However, the genetic heterogeneity and phenotypic variability of HL make it challenging to precisely identify causative variants and genes using NGS (Azaiez et al. 2018; DiStefano et al. 2019).

Although extensive attempts at genetic diagnoses have mainly focused on congenital or pre-lingual HL, including newborn screening for hereditary deafness (Dai et al. 2019; Shearer et al. 2019; Wang et al. 2019), genetic investigations in prospective adult-onset HL patient cohorts appear to be less definitive and more sophisticated owing to other confounding etiologies, such as noise-induced HL and presbycusis (Cunningham and Tucci 2017). For post-lingual HL, the audiogram serves as one of the most widely used phenotypic evaluation tools for HL and can provide decisive clues, particularly in terms of genotype–phenotype correlations (Taylor et al. 2013). Because a specific subset of genes determines hearing frequencies affected in various hearing impairments, physicians can predict the causative genes that correspond to certain types of audiograms with affected frequencies. Interestingly, the ski-slope audiogram pattern, which refers to a steeply down-sloping audiogram pattern with a high threshold observed for sounds with particularly high frequencies, is reportedly associated with pathogenic variants in *TMPRSS3* (MIM 605511) in the autosomal recessive mode (DFNB8/10, MIM 601072) (Chung et al. 2014). Because post-lingual ski-slope HL is one of the most common HL types and patients commonly neglect the chances for diagnosis and treatment, the identification of genetic components for ski-slope HL is essential for both diagnostic and prognostic purposes.

Herein, we aimed to investigate the real-world molecular diagnostic rates of post-lingual HL according to audiogram patterns using a two-track strategy of gene panel and exome sequencing (ES) analyses. Furthermore, additional efforts were made to elucidate the genetic factors involved in ski-slope HL, identify the effects of delta-like ligand 1 (*DLL1*, MIM: 606582) variants, and determine the roles of Notch signaling in ski-slope HL.

## Methods

### Patients and panel/exome sequencing

In total, 192 families with post-lingual non-syndromic HL included in the Yonsei University Hearing Loss (YUHL) cohort were prospectively enrolled from January 2016 until June 2020 (Jung et al. 2017). The participants were enrolled in the YUHL cohort based on the following inclusion criterion: HL without congenital cytomegalovirus infection or other medical diseases primarily affecting hearing function. All individuals registered in the YUHL cohort were referred to Severance Hospital for further evaluation and treatment. Based on pure-tone audiogram patterns, we selected the ski-slope HL group; individuals in this group had audiograms with normal thresholds at low frequencies, but abruptly high thresholds at high frequencies. We defined ski-sloping HL as having a PTA_4_ (pure-tone average at 500, 1000, 2000, 4000 Hz) greater than or equal to 25 dB and PTA_high_ to PTA_low_ greater than or equal to 25 dB (PTA_low_, pure-tone average at 250, 500, and 1000 Hz; PTA_high_, pure-tone average at 2000, 3000, and 4000 Hz). Other individuals with audiogram patterns including flat, reverse, or fluctuating types were grouped into a separate HL group (hereafter, referred to the other HL group).

For molecular genetic testing, genomic DNA was extracted from leukocytes of whole blood samples using a QIAamp Blood DNA mini kit (Qiagen, Hilden, Germany) according to the manufacturer’s instructions. Two-track genetic analysis of panel sequencing and/or ES was performed according to the patients’ willingness for genetic testing when the payment was covered by the national government insurance system. For the customized NGS panel, we selected 207 HL causative genes with validated evidence based on an extensive literature review, the Hereditary Hearing Loss homepage (https://hereditaryhearingloss.org/), Deafness Variation Database (http://deafnessvariationdatabase.org/), and the Online Mendelian Inheritance in Man (OMIM) database (http://www.ncbi.nlm.nih.gov/omim; listed in Supplementary Table S1). For ES, pre-capture libraries were constructed using an Agilent SureSelect V5 enrichment capture kit (Agilent Technologies) according to the manufacturer’s sample preparation protocol. Pooled libraries were sequenced using a MiSeq sequencer (Illumina, San Diego, CA, USA) with the MiSeq Reagent Kit v2 (300 cycles).

### Ethics statement

This study was approved by the institutional review board of Severance Hospital, Yonsei University Health System (IRB #4-2015-0659). We obtained written informed consent from individuals with HL for their participation in this study and the publication of their clinical data.

### Identification of pathogenic variants in known HL genes for genetic diagnosis

Data analysis was performed using CLC Genomic Workbench (version 9.0.1) software (Qiagen) and our custom pipeline. Briefly, raw sequence data were mapped to GRCh37 (hg19) using the Burrows-Wheeler Aligner algorithm, followed by the removal of duplicate reads, realignment of insertions and deletions, base quality recalibration, and variant calling using the Genome Analysis Toolkit. Quality metrics were calculated for each sample using FastQC software and TEQC package. Average read depth and coverage percentages were appropriate for further downstream analysis as quality control indices for NGS in the panel/ES results (Supplementary Table S2). Chromosomal copy number variations were detected using the EXCAVATOR version 2.2 and ExomeDepth version 1.1.10 tools with default settings. Identified variants were annotated, filtered, and evaluated for their pathogenicity using the following databases for pathogenicity evaluation: (1) gnomAD, Exome Aggregation Consortium, Exome Sequencing Project, dbSNP, 1000Genome, and Korean Reference Genome Database for allele frequency; (2) in silico prediction algorithms, including Sorting Tolerant from Intolerant, Polymorphism Phenotyping v2, MutationTaster, Condel, CADD, and REVEL scores; and (3) ClinVar, Deafness Variation Database, and Human Gene Mutation Database (HGMD professional v2020.4.). Final decisions on genetic diagnosis status in every patient were made by a board of genetic HL professionals, including clinicians, otolaryngologists, geneticists, laboratory personnel, and bioinformaticians, through review and discussion in a monthly consensus meeting, in accordance with the HL-specified ACMG guidelines (Oza et al. 2018).

### Clinical characteristics of the HL phenotype in association with genetic diagnosis

To discover the association between HL phenotype and genetic diagnosis status, clinical characteristics of HL were comprehensively evaluated for all patients. Age of HL onset was categorized by early/mid/late decades. Deafness duration, which could also be termed as the lag time for genetic tests, was defined as the period between age of HL onset and age at genetic test. Vestibular symptoms, including spinning sensation and gait imbalance, were thoroughly tested by professional otologists. Detailed genetic counseling was performed by a clinical geneticist to inquire about family history and inheritance mode. From the family history analysis, simplex families included patients without any other affected family members, whereas multiplex families included those having multiple HL patients in a single pedigree.

### Candidate gene prioritization strategy

From genetically undiagnosed cases with ski-slope HL, candidate genes with rare variants (i.e., variants with gnomAD minor allele frequencies lower than 0.1%/0.6% for genes with dominant/recessive inheritance modes, respectively) (Rim et al. 2019) from more than two families were prioritized based on gene-by-gene, variant-level, and affected individual-based analyses. For gene-by-gene evaluation, we determined cumulative allele frequencies for loss-of-function (LoF) variants of specific genes from the gnomAD database (Karczewski et al. 2020). The International Mouse Phenotyping Consortium (IMPC) database (Dickinson et al. 2016) was searched for abnormal results in auditory brainstem response of mouse models to identify candidate genes. For variant-level analysis, the types and locations of variants with different protein domains were considered for prioritization. Affected individual-based analysis included identification of the progressive nature of HL and an inheritance pattern in the pedigree of individuals with ski-slope HL.

### In vitro experimental functional analysis for candidate gene variants

For functional analysis of variants in candidate genes, surface biotinylation, coculture assay, real-time polymerase chain reaction (PCR), and western blotting were performed for in vitro evaluations. Human embryonic kidney 293 T (HEK 293 T) and HeLa cells were cultured in Dulbecco’s modified essential medium (DMEM) supplemented with 10% fetal bovine serum and penicillin (50 IU/mL)/streptomycin (50 μg/mL; Invitrogen, Carlsbad, CA, USA). Cells were transfected with wild-type (WT) or mutant DLL1 plasmids using Lipofectamine and PLUS reagent (Thermo Fisher Scientific, Waltham, MA, USA) according to the manufacturer’s instructions. Surface biotinylation was assessed using 0.3 mg/mL EZ-Link Sulfo-NHS-SS-Biotin and NeutrAvidin (Thermo Fisher Scientific). Cells were homogenized in lysis buffer containing 50 mM Tris (pH 7.4), 1% NP40, 150 mM NaCl, and 1 mM ethylenediaminetetraacetic acid (EDTA) supplemented with a protease inhibitor mixture (Roche). For coculture experiments, HEK 293 T cells transfected with a human NOTCH1 expression plasmid (AddGene) were cocultured with HEK 293 T cells transfected with WT or mutant DLL1 plasmids 24 h prior to coculture. Experiments were performed within 24–36 h after coculture. For real-time PCR, RNA samples were isolated with RiboEx (GeneAll Biotechnology) and reverse-transcribed with an iScript cDNA Synthesis Kit (Bio-Rad Laboratories, Hercules, CA, USA). Samples were assayed with SYBR Green Ready Master Mix (Takara Bio, Shiga, Japan) and appropriate primers (Supplementary Table S3) and analyzed using the StepOnePlus Real-Time PCR System (Applied Biosystems, Foster City, CA, USA). The relative RNA expression levels were calculated using the comparative threshold cycle (Ct) method, and glyceraldehyde 3-phosphate dehydrogenase was used as a control.

### Immunofluorescence of mature murine cochlea samples

Isolated cochlea samples from adult mice (C57BL/6 strain, 7 weeks) were obtained via microdissection. Tissues were fixed by submersion in 4% formaldehyde at 4 °C overnight. After washing twice with phosphate-buffered saline (PBS), the fixed temporal bones were decalcified for 24 h in 10% EDTA/PBS. For paraffin sectioning, serial dehydration of the tissues was performed with ethanol and xylene. The paraffin blocks were sliced into 5-μm-thick sections in the mid-modiolar plane using a microtome (Leica Biosystems). Deparaffinization was performed on the sections, as well as the whole-mounted tissues, with a series of washes with xylene, ethanol, and PBS. The tissues were blocked with 10% donkey serum and incubated with target-specific primary and secondary antibodies (Supplementary Table S4) at 4 °C overnight. The samples were then mounted with mounting solution (Sigma-Aldrich, St. Louis, MO, USA) and imaged using an LSM780 confocal microscope (Zeiss).

## Results

### Differential diagnostic rates of post-lingual HL by known genes according to audiogram patterns

**Fig. 1:**
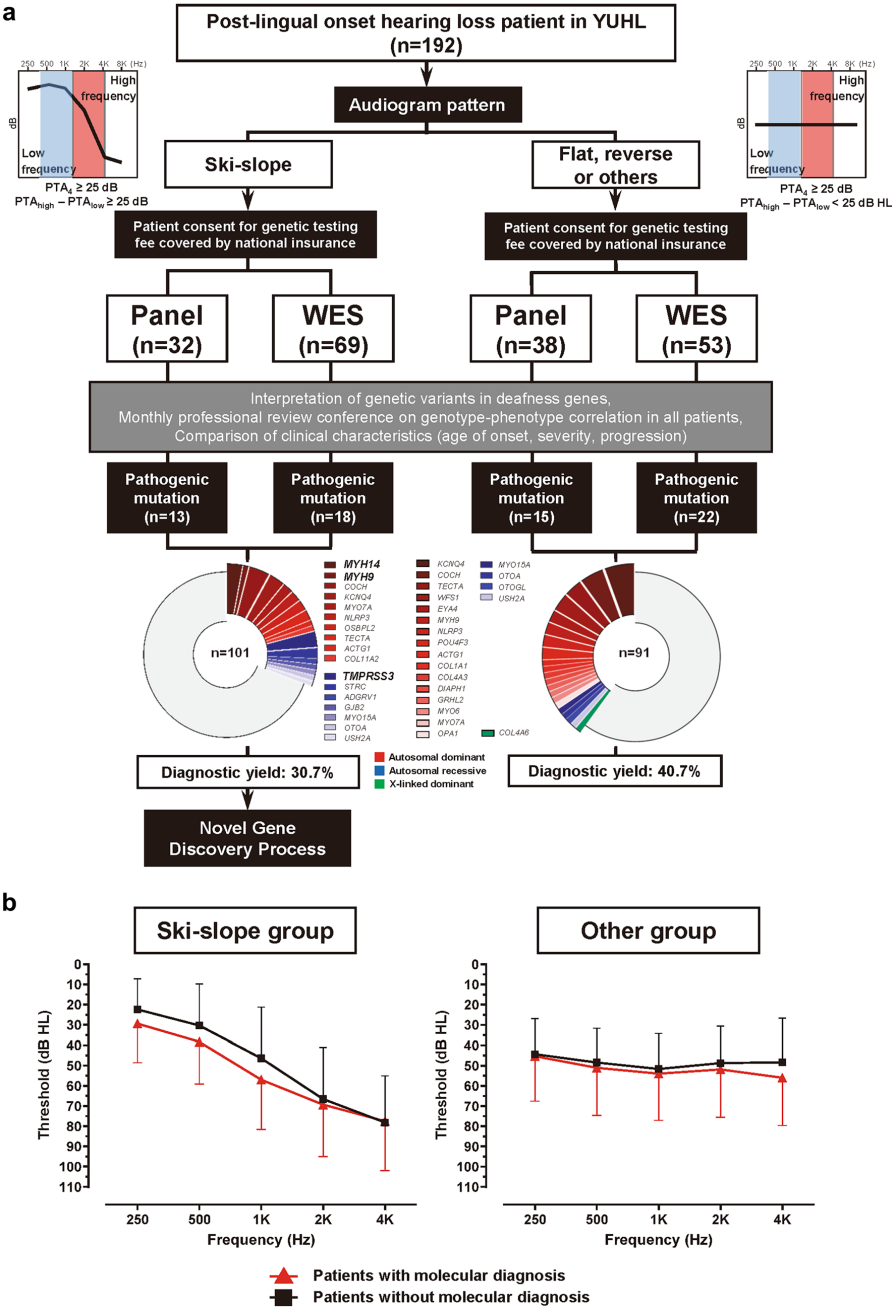
Schematic workflow of genetic analysis in a prospective cohort of patients with post-lingual hearing loss and novel gene discovery in ski-slope hearing loss.** a** 192 families with post-lingual hearing loss were grouped into two groups, i.e., those with ski-slope audiogram patterns and others, and were subjected to genetic analysis via panel/exome sequencing. Genetic heterogeneity was observed in both the ski-slope hearing loss and other hearing loss groups. **b** Audiogram patterns presented by average frequency-specific dB thresholds were similar regardless of genetic diagnosis states in both groups

**Fig. 2:**
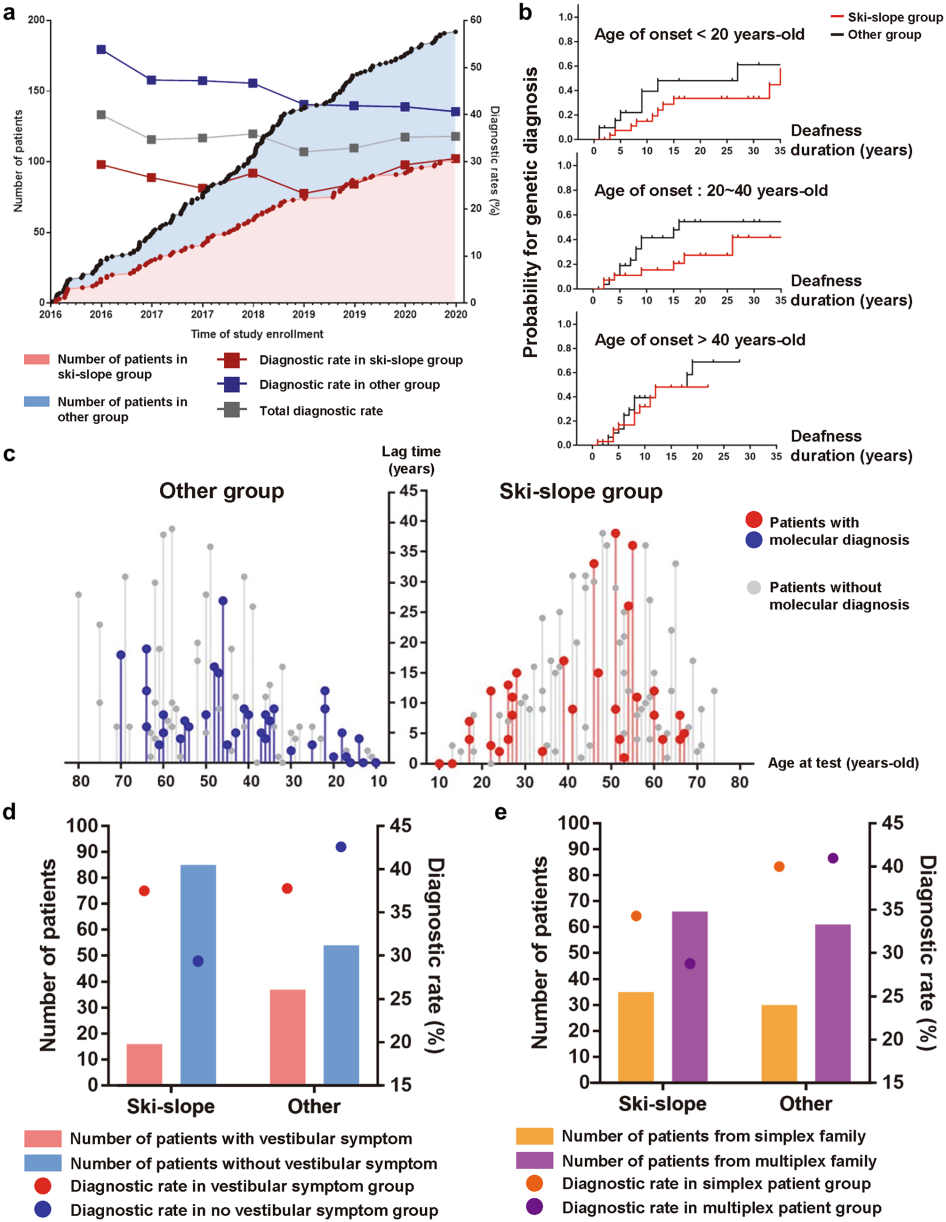
Clinical characteristics of hearing loss according to audiogram pattern and genetic diagnosis status in prospective post-lingual hearing loss cohort. **a** Chronological patterns of enrolled patient numbers and diagnostic rates of panel/exome sequencing according to the audiogram pattern. **b** Differences in probability for genetic diagnosis according to deafness duration between ski-slope and other groups by subgroups with different age of onset for hearing loss. **c** Distribution of lag time until genetic test by age at test in diagnosed (colored line) and undiagnosed (grey line) patients in ski-slope and other groups. **d** Number of patients and diagnostic rates according to accompanied vestibular symptoms in in ski-slope and other groups. **e** Number of patients and diagnostic rates among patients from simplex/multiplex family in ski-slope and other groups

Among 192 families with post-lingual HL included in this prospective study, the initial audiogram pattern analysis identified 101 patients (52.6%) with ski-slope HL (Fig. 1a). One-third of the YUHL cohort (*n* = 70/192) agreed to undergo genetic testing, resulting in gene panel test evaluation. Among 101 families in the ski-slope group, the final diagnostic yield was 30.7% (*n* = 31), including 37 variants in 17 genes. In particular, seven patients had pathogenic variants in three HL genes previously associated with ski-sloping HL (Song et al. 2020), namely, *TMPRSS3*, *MYH14*, and *MYH9* (Supplementary Table S5). By contrast, the final genetic diagnostic yield was 40.7% in the other HL group, which was higher than that in the ski-slope group with consistent statistical significance in the chronological timeline (Figs. 1a, 2a). The relatively high proportions of *TMPRSS3*, *MYH14*, and *MYH9* variants despite lower diagnostic rates in the ski-slope HL group confirmed the suitability of families selected from the YUHL cohort and enabled us to focus on rare genetic variants in the yet-to-be-investigated families.

### Association of HL phenotypes according to genetic diagnosis status

The audiogram patterns in both the ski-slope group and other HL group did not present differences in terms of overall patterns and frequency-specific thresholds according to the genetically diagnosed status (Fig. 1b). When patients with post-lingual HL were classified into three subgroups according to age of onset, the probability for genetic diagnosis by known HL genes was significantly lower for the ski-slope group than for the other HL group in patients with HL onset under 40 years (Fig. 2b, Supplementary Fig. S1a, b). Furthermore, the ski-slope HL group showed a longer lag time until genetic testing in diagnosed and undiagnosed patients compared with the other HL group (*p* = 0.021; Fig. 2c, Supplementary Fig. S1c), suggesting delays in both genetic testing and diagnosis in the ski-slope group. Although the ski-slope HL group showed a significantly lower prevalence of vestibular symptoms (*p* < 0.001), the presence of vestibular symptoms did not lead to higher diagnostic rates in either the ski-slope group or the other HL group (*p* = 0.098; Fig. 2d). By contrast, the diagnostic rates of simplex families were substantially lower in the ski-slope HL group than in the other HL group (Fig. 2e).

### Selection of the candidate gene, *DLL1*

**Fig. 3:**
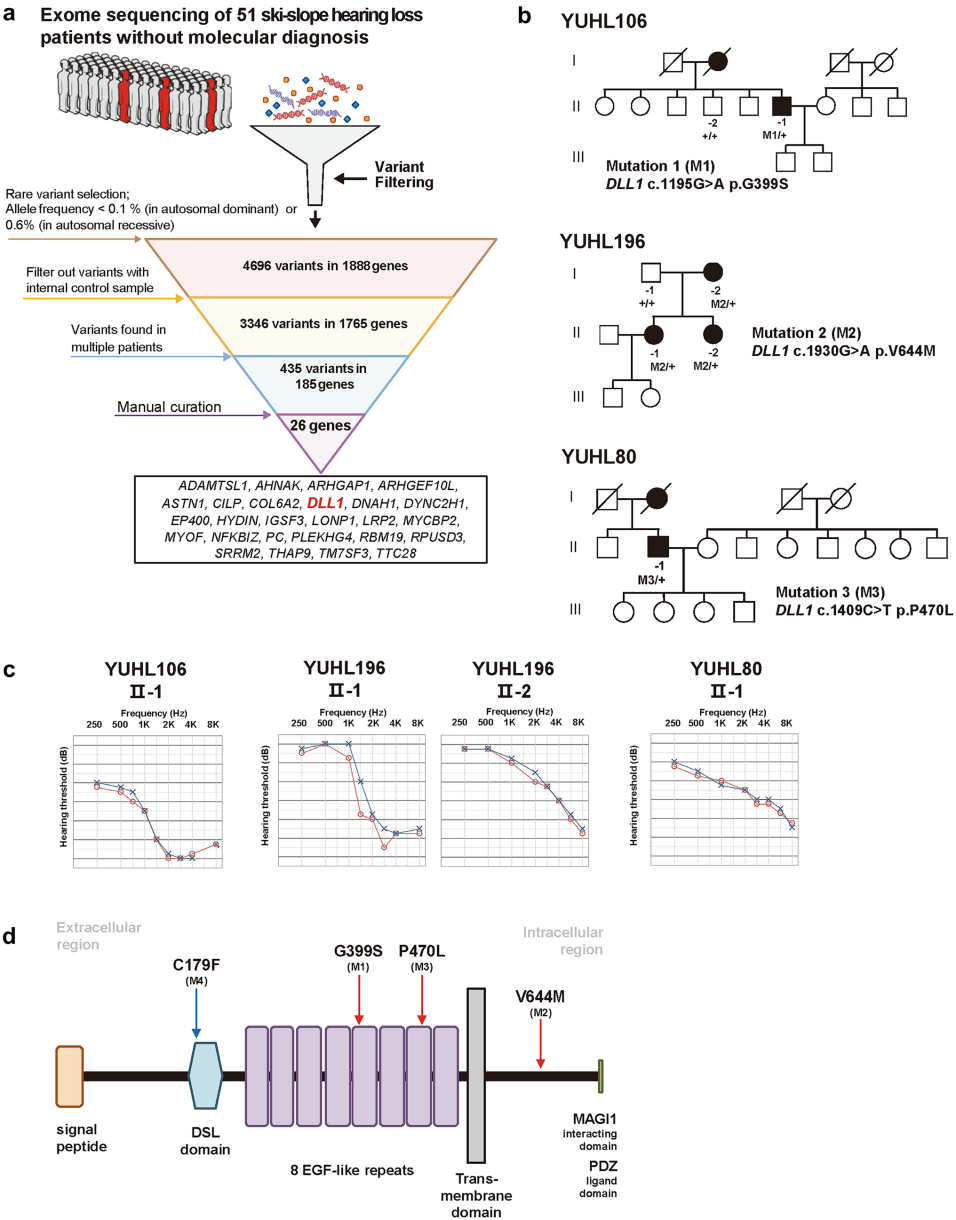
Selection of novel candidate genes from the undiagnosed ski-slope hearing loss group and clinical characteristics of patients with *DLL1* variants.** a** Filtering variants from the exome sequencing of a cohort with 51 patients with undiagnosed ski-slope hearing loss for candidate gene prioritization. **b** Pedigrees and segregation analysis results of three families exhibiting *DLL1* variants. **c** Audiograms of patients with *DLL1* variants exhibiting the ski-slope pattern. **d** Locations of four *DLL1* variants, including one additional missense variant (c.536G>T [p.C179F, M4]) linked to a neurodevelopmental disorder

Based on the overall diagnostic rate of 35.4% (68/192) in post-lingual HL by currently validated deafness-associated genes, further analysis of ES from the 51 genetically undiagnosed patients in the ski-slope HL group enabled the identification of 4696 rare variants with allele frequencies less than 0.001 or 0.006, based on their inheritance patterns (Rim et al. 2019); these were further filtered out by comparing their values with those of healthy control samples (Fig. 3a). After manually curating genes with rare variants in multiple individuals, 26 candidate genes were prioritized, based on our evidence, which included details, such as gene features, variant analyses, and clinical characteristics (Supplementary Table S6). *DLL1* (MIM 606582)*,* which encodes delta-like 1, was the top scoring candidate gene, based on evidence obtained after analyzing the following aspects: (1) gene-by-gene analysis: no LoF variant was observed in gnomAD, a deaf mouse model in IMPC, and publications on cochlear epithelial expression in PubMed; (2) variant-level analysis: three deleterious missense variants (i.e., c.1195G>A [p.G399S, M1], c.1930G>A [p.V644M, M2], and c.1409C>T [p.P470L, M3]) were observed at evolutionarily conserved regions (Supplementary Table S7); and (3) all variants were segregated in affected members of three unrelated families with autosomal dominant inheritance and ski-slope audiogram (Fig. 3b, c).

### Variants in *DLL1* conferred Notch signaling gain-of-function in vitro

To investigate the effects of missense variants detected in the ski-slope HL group, we first examined membrane expression levels of four DLL1 variants (Fig. 3d), including another *DLL1* missense variant (c.536G>T [p.C179F, M4]), which was recently reported to be responsible for a congenital neurodevelopmental disorder (MIM 618709) with an autosomal dominant inheritance (Fischer-Zirnsak et al. 2019). Pathogenic *DLL1* variants linked to the neurodevelopmental disorder were predicted to exhibit the LoF effect; indeed, the *DLL1* p.C179F (M4) variant was trapped intracellularly, which revealed the presence of a trafficking defect (Supplementary Fig. S2a). By contrast, all three *DLL1* variants (i.e., p.G399S [M1], p.V644M [M2], p.P470L [M3]) identified in this study were expressed in the membrane in the same manner as that of wild-type (WT) DLL1 (Supplementary Fig. S2a). Interestingly, the surface biotinylation experiment revealed that the membrane expression levels of *DLL1* variants p.V644M and p.P470L were increased, and the incremental values were statistically significant compared with those for WT *DLL1* (Supplementary Fig. S2b, c). Although the membrane expression levels of p.G399S did not show a significant increase, CUPSAT (Parthiban et al. 2006), one of the most widely used protein stability prediction tools, proposed the increased stability for p.G399S (ΔΔG = 0.98) and reduced stability for p.C179F (ΔΔG = − 7.49).

**Fig. 4:**
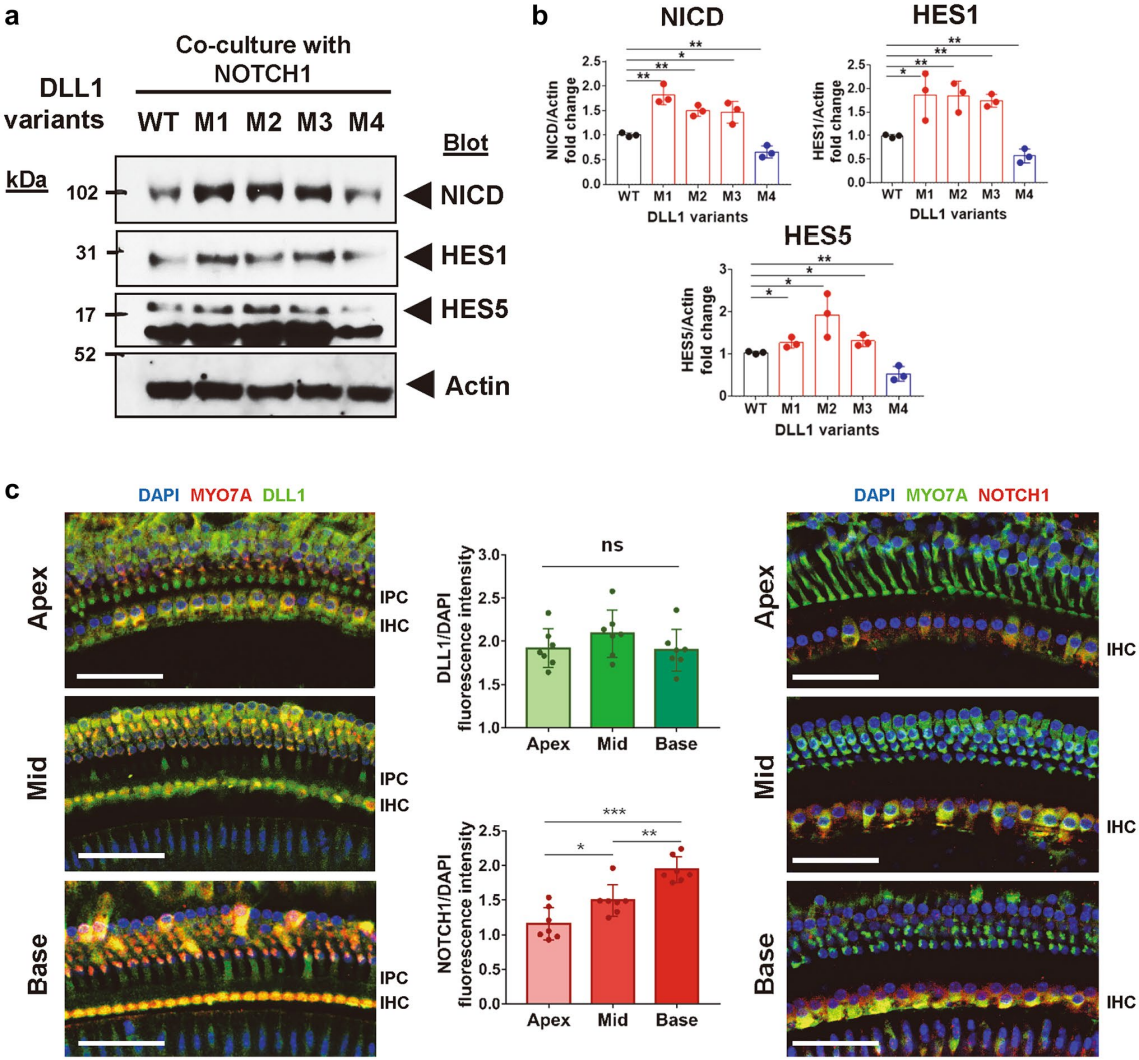
Functional in vitro and in vivo experiments suggesting gain-of-function effects of DLL1 variants and deleterious effects of Notch signaling overactivation on the mature cochlea. **a**, **b** Immunoblotting results of downstream markers of Notch signaling activation (NICD, HES1, and HES5) were obtained using a coculture assay to analyze Notch signaling activation by wild-type and variant DLL1 (Supplementary Fig. S3a). **c** The expression levels of DLL1 (left panel) and NOTCH1 (right panel) according to tonotopy, from the apex to base in whole-mount samples of cochlear tissues from 7-week-old mice and their quantification; the fluorescence levels were normalized with the DAPI fluorescence intensity values. The data represent the means ± standard errors of the means. *p < 0.05, **p < 0.01, ***p < 0.001. Scale bars: 20 μm (**c**). *DIC* differential interference contrast microscopy images

Next, we investigated the effects of *DLL1* variants on Notch signaling via coculture assays (Supplementary Fig. S3a). Experimental feasibility for Notch activation levels in coculture assays and for appropriate antibodies enabled us to exclude the effects of endogenous NOTCH1 and/or DLL1 expression (Supplementary Fig. S3b–d). When cocultured with NOTCH1-expressing cells, the expression levels of the Notch intracellular domain (NICD), as well as *HES1* (MIM 139605) and *HES5* (MIM 607348), two target genes of NICD (Zine et al. 2001), were increased in all three variants (M1, M2, and M3), compared with those with WT *DLL1*, suggesting that these variants caused gain-of-function Notch activation (Fig. 4a, b). Interestingly, the M4 variant, which was predicted to result in an LoF effect in a previous publication (Fischer-Zirnsak et al. 2019), caused the downregulation of Notch signaling, compared with expression with WT *DLL1* (Fig. 4a, b). Furthermore, the activation or downregulation of Notch signaling via changes in mRNA were within the twofold range in real-time PCR experiments (Supplementary Fig. S3e).

### Expression patterns of DLL1 and NOTCH1 in mature murine cochlea

Although the roles of ligands causing Notch signaling activation, including DLL1 and Jagged 1/2, have been elucidated in cochlear development processes for the accurate patterning of auditory hair cell differentiation, it is unclear whether Notch signaling is involved in homeostasis in the adult cochlea (Takebayashi et al. 2007; Zine et al. 2000). To explore this aspect, DLL1 and NOTCH1 expression patterns were examined via immunofluorescence analysis in mature murine cochlea (Supplementary Fig. S4a–c). In cochlea from 7-week-old mice, DLL1 was expressed in supporting cells, including inner phalangeal cells, pillar cells, and Deiter’s cells (Supplementary Fig. S4a, c), whereas NOTCH1 was strongly expressed in the inner hair cells and weakly expressed in the outer hair cells (Supplementary Fig. S4b, c). By contrast, DLL1 and NOTCH1 were mainly expressed in the hair and supporting cells, respectively, in the embryonic and postnatal cochlea (Supplementary Fig. S4d–f), consistent with the results of previous reports (Basch et al. 2016; Zine et al. 2000). To investigate DLL1 and NOTCH1 expression in a tonotopy gradient, fluorescence intensities normalized to those of 4′,6-diamidino-2-phenylindole (DAPI) were measured in whole mount samples. While DLL1 expression levels were similar across all areas (Fig. 4c), NOTCH1 expression levels were lowest at the apex and highest at the base, indicating a gradient pattern with statistical significance (Fig. 4c). From these data, we observed that NOTCH1 expression was gradually decreased in hair cells from the basal turn, tuned at high frequencies, to the apical turn; this might be relevant to HL vulnerabilities at high frequencies.

## Discussion

Various genetic studies on HL using panel sequencing and/or ES previously evaluated clinical efficiency according to ethnicity, age of onset, or severity (Kim et al. 2020; Shearer et al. 2014; Yuan et al. 2020). However, few studies have investigated the diagnostic rates of genetic testing in relation to audiogram patterns (Song et al. 2020), which is the first-line diagnostic tool in HL evaluation. In our prospective study, the genetic diagnostic rates of patients with post-lingual HL were consistently lower in the ski-slope HL group than in the other HL group. Interestingly, approximately 20% of genetically diagnosed patients carried pathogenic mutations in recessive genes, even though our cohort consisted of patients with post-lingual HL, suggesting a complex relationship between inheritance mode and age of onset. Moreover, the ski-slope HL group showed a lower probability for genetic diagnosis, particularly in patients with early HL onset from simplex families. These results indicated that more efforts are needed for accurate and timely genetic diagnosis of ski-slope HL, despite the longer deafness duration and lag time until genetic testing, potentially related to the milder disease severity.

The additional genetic components responsible for hereditary HL are still unclear, as more than 500 genes are expected to be related to deafness onset (Bowl et al. 2017; Ingham et al. 2019). Although the diagnostic rates based on known pathogenic variants in patients with post-lingual HL reached similar yields compared with those with pre-lingual and severe HL (Sheppard et al. 2018), genetic delineation focusing on the specific phenotype of the ski-slope audiogram pattern resulted in a lower diagnostic rate and, therefore, a higher chance for novel candidate gene discovery. In particular, diseases or even disease subtypes with lower rates of genetic diagnosis based on the currently known genetic landscape require a more extensive genetic workup for the identification of novel candidate genes and subsequent functional validations, despite limitations in currently available clinical ES, which may not be able to identify other pathogenic noncoding or epigenetic variants.

Based on our functional study results, individuals born with germline missense gain-of-function variants in *DLL1* may not exhibit defects as severe as those with other genetic diseases, owing to LoF variants in genes involved in Notch signaling. However, Notch signaling overactivation was induced in individuals with rare *DLL1* missense variants compared with that in individuals without *DLL1* variants under physiological conditions, possibly resulting from acoustic trauma and senescence. As the cumulative effects of Notch signaling overactivation increased during the adulthood, these patients remained susceptible to post-lingual ski-slope HL because of the deleterious effects of *DLL1* variants. The gradient expression pattern of NOTCH1 receptors revealed in this study may contribute to high frequency-specific hearing impairments. Furthermore, the post-lingual phenotype may also be associated with the incomplete penetrance of *DLL1* missense variants. Indeed, the relatively high allele frequency in the gnomAD database (particularly for p.P470L; 0.03%) suggested that the effects of certain variants present interindividual variability or that gene–environment or gene–host interactions may affect variant carriers in different ways.

Recently, variations in Notch signaling in mature adult organs have been shown to be closely related to various human diseases, such as liver cirrhosis (Zhu et al. 2018), glucose intolerance (Bartolome et al. 2019), and schizophrenia (Ables et al. 2011). Numerous studies have investigated the roles of Notch signaling during cochlear development and regeneration, evaluating their expression patterns, spatiotemporal dynamics, and regulation (Nandagopal et al. 2018). Interestingly, a recent study on the adult mouse cochlea proved that only the transiently overexpressed NICD and Myc are essential for survival and the ability of supporting cells to undergo transdifferentiation for regeneration (Shu et al. 2019). However, continuously sustained signals of either NICD or Myc are detrimental to hair cell differentiation, because the targets of NICD, i.e., Hes1 and Hes5, are antagonists of Atoh1, which is a key player in hair cell differentiation (Shu et al. 2019; Zheng et al. 2000). Nevertheless, few studies have focused on the associations between germline variants in Notch signaling component genes and altered functions of the mature adult cochlea (Terrinoni et al. 2013). Our results highlight opportunities for the genetic diagnosis of ski-slope HL and the discovery of novel therapeutic agents, consistent with the results of current clinical trials examining Notch signaling inhibitors for adult-onset HL (Andersson and Lendahl 2014) and a proof-of-concept study regarding hair cell regeneration in adult guinea pigs through siRNA *Hes1* modulation (Du et al. 2018), based on the evidence that Notch signaling inhibition promotes differentiation into hair cells in the cochlea (Atkinson et al. 2015; Mizutari et al. 2013).

In conclusion, comprehensive genetic diagnosis using panel sequencing/ES in a prospective cohort of patients with post-lingual HL revealed differential diagnostic rates according to audiogram patterns. In further analyses of the genetically undiagnosed ski-slope audiogram group, *DLL1* missense variants were identified as genetic components resulting from the overactivation of Notch signaling, the degree of which appear to be within the acceptable range during development. Functional studies demonstrated that *DLL1* missense variants resulted in gain-of-function effects, whereas a *DLL1* variant reportedly associated with a neurodevelopmental disorder was proven to be associated with the LoF effect. Notch signaling in the mature adult cochlea is thus altered by genetic variants contributing to the development of ski-slope HL, in combination with aging and acoustic traumas.

## Supplementary Information

Below is the link to the electronic supplementary material.

Supplementary file1 (PDF 5012 KB)

## Data Availability

The data sets generated during and/or analyzed during the current study will be available from the corresponding author on request.
