# Supplementary material for: Differential genetic diagnoses of adult post-lingual hearing loss according to the audiogram pattern and novel candidate gene evaluation
Source: Hum Genet. 2021 Sep 14;141(3-4):915–27. doi: 10.1007/s00439-021-02367-z (PMC9034979; doi:10.1007/s00439-021-02367-z)
Supplement: Supplementary file 1 — Supplementary file1 (PDF 5012 KB) [file 439_2021_2367_MOESM1_ESM.pdf]

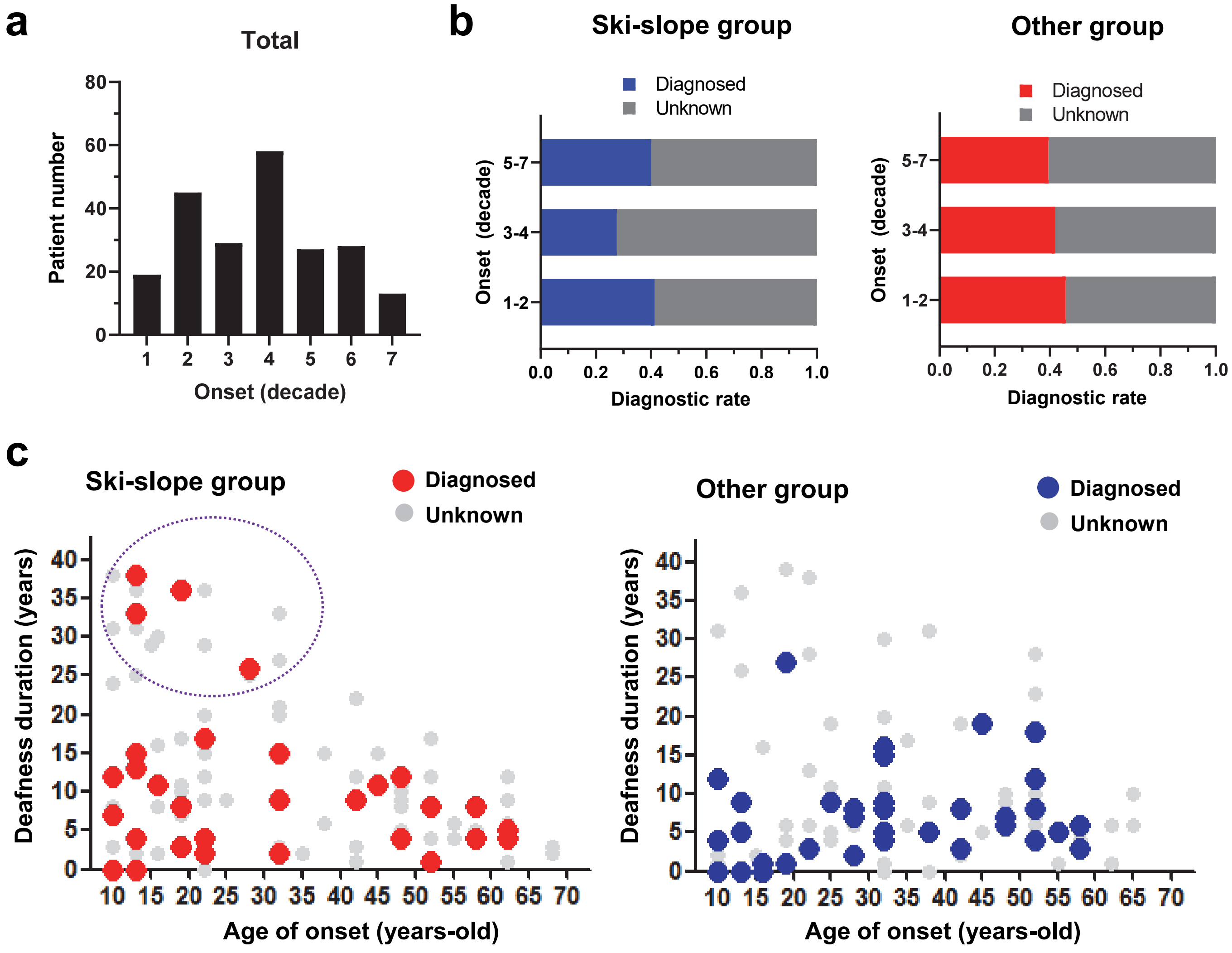


**Supplementary Fig. S1 Age of onset distribution for patients in this study.** **a** Number of patients according to hearing loss onset by decades. **b** Comparison of diagnostic rates according to hearing loss onset between ski-slope group and other group. **c** Deafness duration profiling according to age of onset, ski-slope/other audiogram, and genetic diagnosis status.

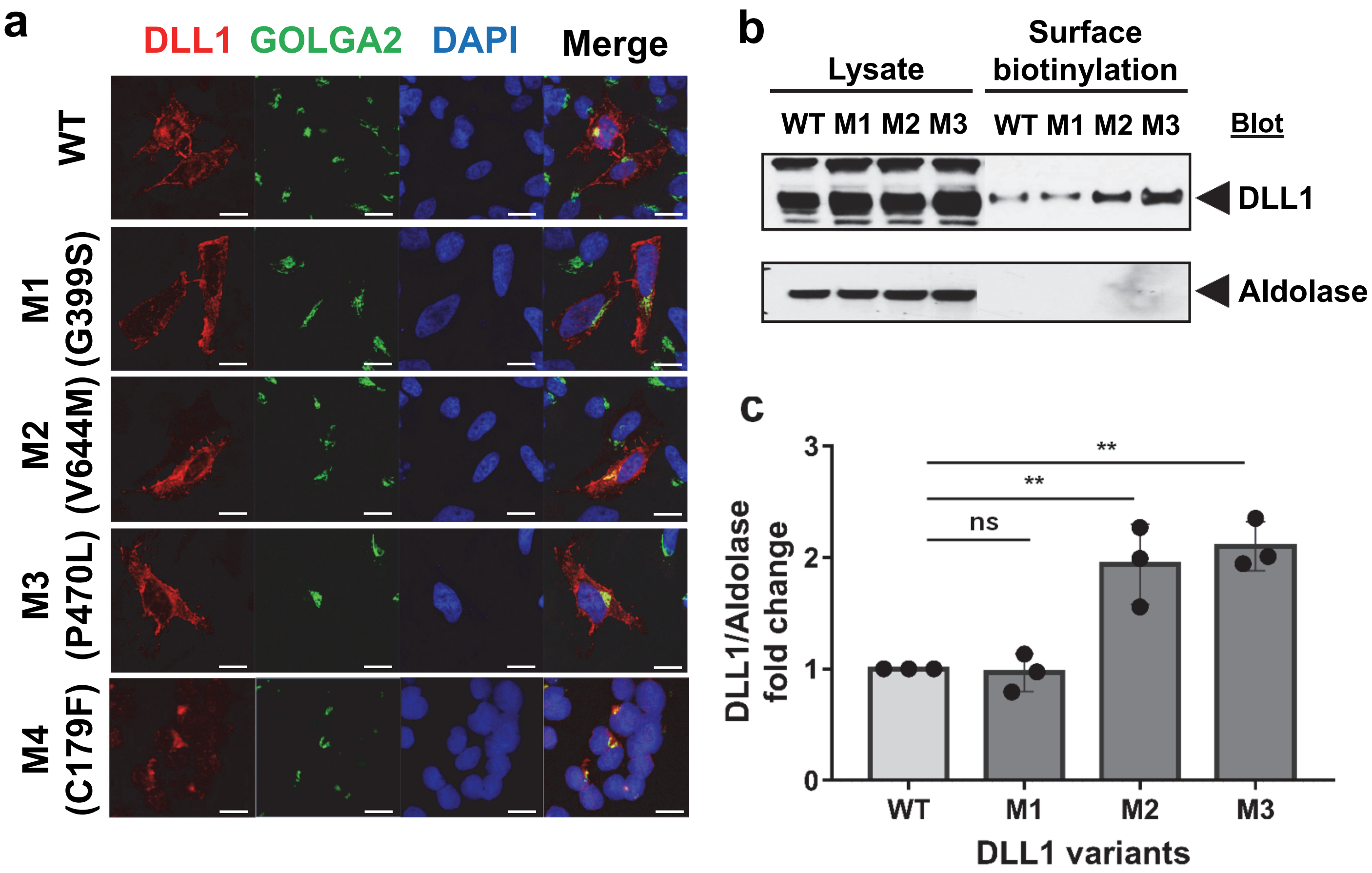


**Supplementary Fig. S2 Gain-of-function effects of *DLL1* variants on Notch signaling activation.** **a** Determination of the expression patterns of wild-type and variant DLL1 in HeLa cells via immunofluorescence staining. Golgin A2 (GOLGA2) was used as a Golgi marker. **b, c** Surface biotinylation assays was performed to quantify the surface expression levels of three DLL1 variant proteins, compared to that of wild-type DLL1. Scale bars: 20 μm (**a**).

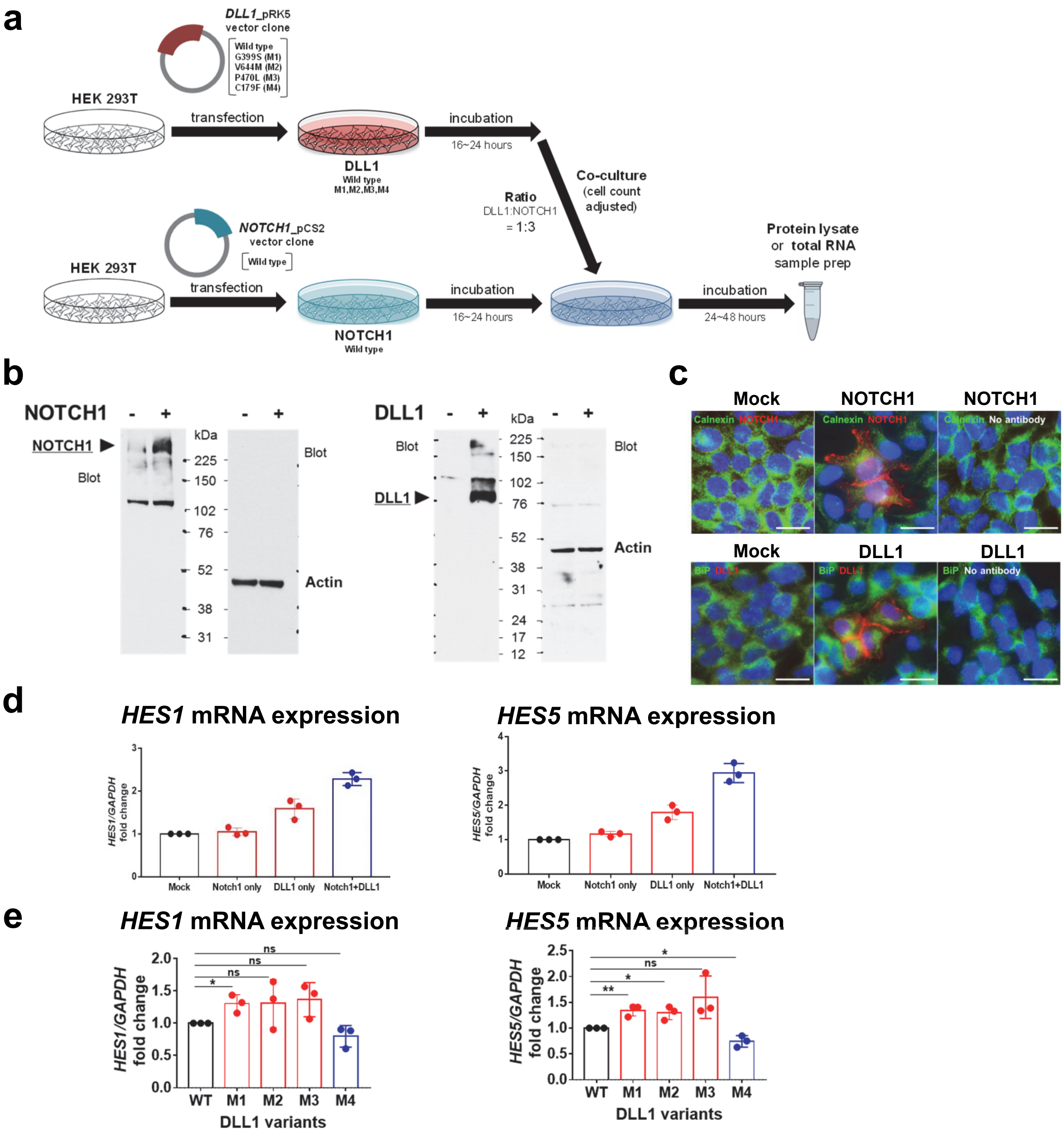


**Supplementary Fig. S3 Coculture assay validation for evaluation of *DLL1* variants on Notch signaling activation.** **a** Experimental workflow of a coculture assay used to analyze Notch signal activation of four DLL1 variants. **b, c** Validation for overexpression of NOTCH1 and wild-type DLL1 plasmids with appropriate antibodies by immunoblotting (**b**) and immunofluorescence staining (**c**). **d** Validation for Notch signaling activation using coculture assay by real-time PCR. **e** Real-time PCR was performed to quantitatively compare HES1 and HES5 mRNA expression levels from the co-culture samples used as described in (**a**). ns, not significant; *p<0.05, **p<0.01; scale bars: 40 μm (**c**).

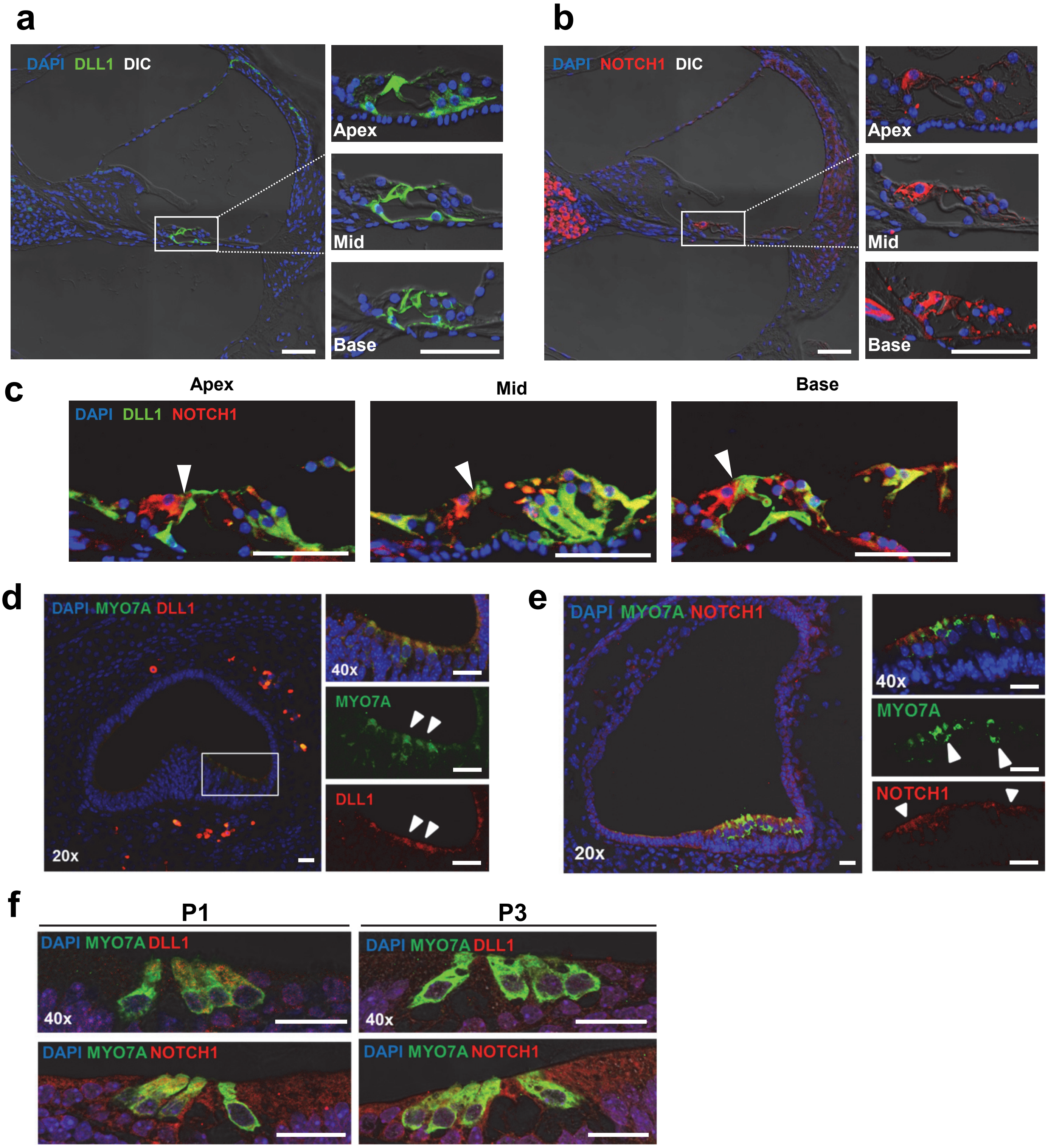


**Supplementary Fig. S4 Expression of DLL1 and NOTCH1 in mature and embryonic murine cochlea.** **a** Cross-section of a sample of pillar cells obtained from a 7-week-old mature murine cochlea exhibiting DLL1 expression. **b** Cross-section of a sample of inner hair cells obtained from a 7-week-old mature murine cochlea exhibiting NOTCH1 expression. **c** Interaction of DLL1 and NOTCH1 expression of 7-weeks-old mature murine cochlea in cross-section by co-immunostaining. **d, e** The expression levels of DLL1 (**d**) and NOTCH1 (**e**) in a whole-mount sample of embryonic (E14.5-16.5) immature murine cochlea. **f** The expression levels of DLL1 and NOTCH1 in cross-section samples of postnatal (P1, P3) immature murine cochlea. Scale bars: 50 μm (**a-c**) and 20 μm (**d, e, f**).

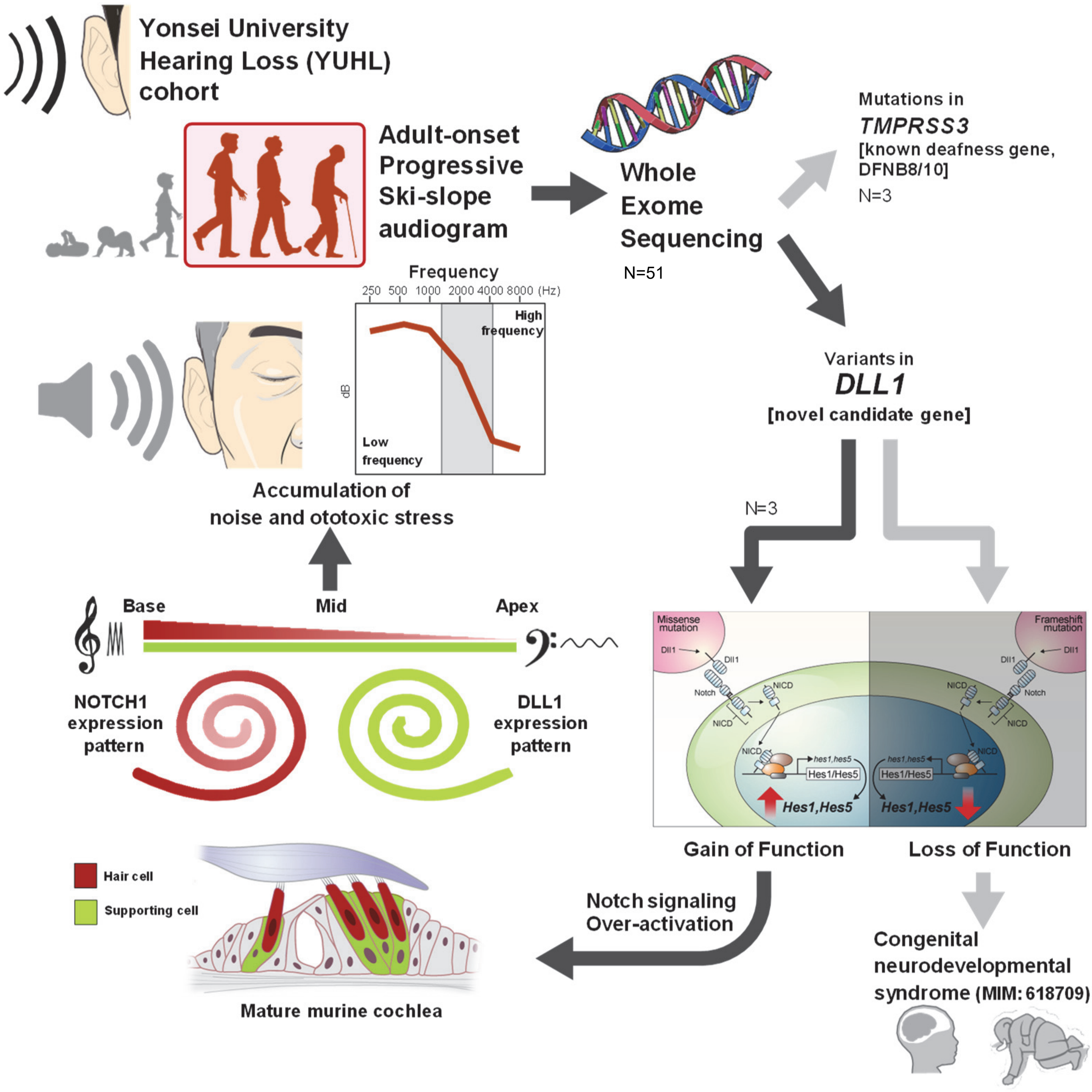


**Supplementary Fig. S6 Graphical abstract.** Individuals with rare missense variants in *DLL1* are susceptible to present adult-onset ski-slope hearing loss because of the deleterious effects of *DLL1* variants, which result in the over-activation of Notch signals, possibly cued by acoustic trauma and senescence.

**Supplementary Table S1. A list of 207 known hearing loss-related genes evaluated in the gene panel in this study.**

| # | Gene | OMIM number | Mode | Detailed description in OMIM (matched phenotype MIM number) | HHL | DVD | Reference 1 (2016) | Reference 2 (2018)_AUDIOME | Reference 3 (2018)_OtoGenome |
|---|---|---|---|---|---|---|---|---|---|
| 1 | *ABHD12* | 613599 | AR | Y (612674) | | Y | Evidential level 3 | Y | |
| 2 | *ABHD5* | 604780 | AR | Y (275630) | | | | | |
| 3 | *ACTB* | 102630 | AD | Y (243310) | | Y | Evidential level 3 | | |
| 4 | *ACTG1* | 102560 | AD | Y (604717) | Y | Y | Evidential level 3 | Y | Y (category 2) |
| 5 | *ADCY1* | 103072 | AR | Y (610154) | Y | Y | | | Y (category 3) |
| 6 | *ADGRV1* | 602851 | AR | Y (605472) | Y | Y | | Y | Y (category 1) |
| 7 | *AIFM1* | 300169 | XR | Y (300614) | Y | Y | Evidential level 2 | | |
| 8 | *ALMS1* | 606844 | AR | Y (203800) | | Y | Evidential level 3 | Y | Y (category 1) |
| 9 | *ANKH* | 605145 | AD | Y (123000) | | Y | Evidential level 3 | | |
| 10 | *ARSB* | 611542 | AR | Y (253200) | | | | | |
| 11 | *ATP2B2* | 108733 | AR | Y (601386) | | Y | Evidential level 1 | | |
| 12 | *ATP6V1B1* | 192132 | AR | Y (267300) | | Y | Evidential level 3 | Y | Y (category 1) |
| 13 | *BCS1L* | 603647 | AR | Y (262000, 124000) | | Y | Evidential level 3 | Y | Y (category 2) |
| 14 | *BDP1* | 607012 | AR | Y (618257) | Y | Y | | | |
| 15 | *BSND* | 606412 | AR | Y (602522) | Y | Y | Evidential level 3 | Y | Y (category 1) |
| 16 | *BTD* | 609019 | AR | Y (253260) | | Y | | | |
| 17 | *CABP2* | 607314 | AR | Y (614899) | Y | Y | Evidential level 1 | Y | Y (category 3) |
| 18 | *CACNA1D* | 114206 | AR | Y (614896) | | Y | Evidential level 2 | Y | Y (category 3) |
| 19 | *CATSPER2* | 607249 | AR | | | | Evidential level 3 | | Y (category 2) |
| 20 | *CCDC50* | 611051 | AD | Y (607453) | Y | Y | Evidential level 2 | Y | Y (category 2) |
| 21 | *CD151* | 602243 | AR | Y (609057) | | | Evidential level 1 | | |
| 22 | *CD164* | 603356 | AD | Y (616969) | Y | Y | | | Y (category 3) |
| 23 | *CDC14A* | 603504 | AR | Y (616958) | Y | Y | | Y | Y (category 3) |
| 24 | *CDH23* | 605516 | AR | Y (601386) | Y | Y | Evidential level 3 | Y | Y (category 3) |
| 25 | *CDKN1C* | 600856 | AD | Y (130650, 614732) | | | Evidential level 3 | | |
| 26 | *CEACAM16* | 614591 | AD | Y (614614) | Y | Y | Evidential level 2 | Y | Y (category 1) |
| 27 | *CEP78* | 617110 | AR | Y (617236) | | Y | | Y | Y (category 2) |
| 28 | *CHD7* | 608892 | AD | Y (214800, 612370) | | Y | Evidential level 3 | | Y (category 2) |
| 29 | *CHSY1* | 608183 | AR | Y (605282) | | Y | Evidential level 3 | | |
| 30 | *CIB2* | 605564 | AR | Y (609439) | Y | Y | Evidential level 3 | Y | Y (category 3) |
| 31 | *CISD2* | 611507 | AR | Y (604928) | | Y | Evidential level 2 | Y | Y (category 1) |
| 32 | *CLDN14* | 605608 | AR | Y (614035) | Y | Y | Evidential level 3 | Y | Y (category 3) |
| 33 | *CLDN9* | 615799 | AR | Y (619093) | Y | Y | | | |
| 34 | *CLIC5* | 607293 | AR | Y (616042) | Y | Y | Evidential level 1 | Y | Y (category 3) |
| 35 | *CLPP* | 601119 | AR | Y (614129) | | Y | Evidential level 3 | Y | Y (category 1) |
| 36 | *CLRN1* | 606397 | AR | Y (276902) | Y | Y | Evidential level 3 | Y | Y (category 3) |
| 37 | *COCH* | 603196 | AD | Y (601369) | Y | Y | Evidential level 3 | Y | Y (category 2) |
| 38 | *COL11A1* | 120280 | AD | Y (618533, 604841) | Y | Y | Evidential level 3 | Y | |
| 39 | *COL11A2* | 120290 | AD/AR | Y (601868, 154780, 609706) | Y | Y | Evidential level 3 | Y | Y (category 2) |
| 40 | *COL2A1* | 120140 | AD | Y (132450, 108300) | Y | Y | Evidential level 3 | Y | |
| 41 | *COL4A3* | 120070 | AD/AR | Y (104200, 203780) | Y | Y | Evidential level 3 | Y | Y (category 1) |
| 42 | *COL4A4* | 120131 | AD/AR | Y (141200, 203780) | Y | Y | Evidential level 3 | Y | Y (category 1) |
| 43 | *COL4A5* | 303630 | XD | Y (301050) | Y | Y | Evidential level 3 | Y | Y (category 2) |
| 44 | *COL4A6* | 303631 | XR | Y (300914) | Y | Y | Evidential level 1 | | |
| 45 | *COL9A1* | 120210 | AR | Y (614134) | Y | Y | Evidential level 2 | Y | |
| 46 | *COL9A2* | 120260 | AR | Y (614284) | Y | Y | Evidential level 3 | Y | |
| 47 | *COL9A3* | 120270 | AD | | | Y | Evidential level 3 | Y | |
| 48 | *CRYL1* | 609877 | AD | | | | Evidential level 1 | | |
| 49 | *CRYM* | 123740 | AD | Y (616357) | Y | Y | Evidential level 1 | | |
| 50 | *DCDC2* | 605755 | AR | Y (610212) | Y | Y | | | |
| 51 | DFNA5 | 608798 | AD | Y (600994) | Y | Y | Evidential level 3 | Y | Y (category 2) |
| 52 | DFNB59 | 610219 | AR | Y (610220) | Y | Y | Evidential level 3 | Y | Y (category 2) |
| 53 | *DIABLO* | 605219 | AD | Y (614152) | Y | Y | Evidential level 2 | Y | Y (category 2) |
| 54 | *DIAPH1* | 602121 | AD | Y (124900) | Y | Y | Evidential level 2 | Y | Y (category 3) |
| 55 | *DIAPH3* | 614567 | AD | Y (609129) | | Y | Evidential level 1 | | |
| 56 | *DLX5* | 600028 | AR | Y (220600) | | Y | Evidential level 2 | | |
| 57 | *DMXL2* | 612186 | AD | Y (617605) | Y | Y | | Y | |
| 58 | *DNMT1* | 126375 | AD | Y (604121, 614116) | | Y | | | |
| 59 | *DSPP* | 125485 | AD | Y (605594) | | Y | Evidential level 3 | | |
| 60 | *EDN3* | 131242 | AD/AR | Y (613265) | Y | Y | Evidential level 3 | Y | Y (category 2) |
| 61 | *EDNRB* | 131244 | AD/AR | Y (277580) | Y | Y | Evidential level 3 | Y | Y (category 3) |
| 62 | *ELMOD3* | 615427 | AR | Y (615429) | Y | Y | | | |
| 63 | *EPS8* | 600206 | AR | Y (615974) | Y | Y | | Y | Y (category 1) |
| 64 | *EPS8L2* | 614988 | AR | Y (617637) | Y | Y | | | |
| 65 | *ERCC2* | 126340 | AR | | | | Evidential level 3 | | |
| 66 | *ERCC3* | 133510 | AR | Y (610651) | | | Evidential level 2 | | |
| 67 | *ESPN* | 606351 | AD/AR | Y (609006) | Y | Y | Evidential level 3 | Y | Y (category 1) |
| 68 | *ESRP1* | 612959 | AR | Y (618013) | Y | | | Y | |
| 69 | *ESRRB* | 602167 | AR | Y (608565) | Y | Y | Evidential level 3 | Y | Y (category 1) |
| 70 | *EYA1* | 601653 | AD | Y (166780) | Y | Y | Evidential level 3 | Y | Y (category 3) |
| 71 | *EYA4* | 603550 | AD | Y (601316, 602588) | Y | Y | Evidential level 3 | Y | Y (category 3) |
| 72 | *FAM65B* | 611410 | AR | Y (616515) | Y | Y | | Y | |
| 73 | *FGF3* | 164950 | AR | Y (610706) | | Y | Evidential level 3 | Y | |
| 74 | *FGFR3* | 134934 | AD/AR | Y (149730, 602849, 610474) | | Y | | | |
| 75 | *FOXC1* | 601090 | AD | Y (602482) | | | | | |
| 76 | *GAB1* | 604439 | AR | Y (605428) | Y | Y | | | |
| 77 | *GATA3* | 131320 | AD | Y (146255) | | Y | Evidential level 3 | | |
| 78 | *GIPC3* | 608792 | AR | Y (601869) | Y | Y | Evidential level 3 | Y | Y (category 1) |
| 79 | *GJA1* | 121014 | AD/AR | Y (164200, 218400) | | | Evidential level 3 | | |
| 80 | *GJB2* | 121011 | AD/AR | Y (601544, 220290) | Y | Y | Evidential level 3 | Y | Y (category 1) |
| 81 | *GJB3* | 603324 | AD/AR | Y (612644, 220290) | Y | Y | Evidential level 3 | | |
| 82 | *GJB6* | 604418 | AD/AR | Y (612643, 612645, 220290) | Y | Y | Evidential level 3 | Y | Y (category 2) |

| | | | | | | | | | |
|---|---|---|---|---|---|---|---|---|---|
| 83 | *GPSM2* | 609245 | AR | Y (604213) | Y | Y | Evidential level 2 | Y | Y (category 2) |
| 84 | *GRAP* | 604330 | AR | Y (618456) | Y | Y | | | |
| 85 | *GRHL2* | 608576 | AD | Y (608641) | Y | Y | Evidential level 2 | Y | Y (category 1) |
| 86 | *GRXCR1* | 613283 | AR | Y (613285) | Y | Y | Evidential level 2 | Y | Y (category 1) |
| 87 | *GRXCR2* | 615762 | AR | Y (615837) | Y | Y | | Y | |
| 88 | *GSTP1* | 134660 | AD | | | | | | |
| 89 | *HARS1* | 142810 | AR | Y (614504) | | Y | | | |
| 90 | *HARS2* | 600783 | AR | Y (614926) | Y | Y | Evidential level 2 | Y | Y (category 2) |
| 91 | *HGF* | 142409 | AR | Y (608265) | Y | Y | Evidential level 2 | Y | Y (category 3) |
| 92 | *HOMER2* | 604799 | AD | Y (616707) | Y | Y | | Y | |
| 93 | *HOXB1* | 142968 | AR | Y (614744) | | Y | Evidential level 1 | | |
| 94 | *HSD17B4* | 601860 | AR | Y (233400, 261515) | Y | Y | Evidential level 2 | Y | Y (category 3) |
| 95 | *IFNLR1* | 607404 | AR | | Y | Y | | | |
| 96 | *ILDR1* | 609739 | AR | Y (609646) | Y | Y | Evidential level 3 | Y | Y (category 2) |
| 97 | *JAG1* | 601920 | AD | Y (617992) | | | Evidential level 3 | | |
| 98 | *KARS* | 601421 | AR | Y (613916) | Y | Y | Evidential level 2 | | Y (category 2) |
| 99 | *KCNE1* | 176261 | AR | Y (612347) | Y | Y | Evidential level 2 | Y | Y (category 2) |
| 100 | *KCNQ1* | 607542 | AR | Y (220400) | | Y | Evidential level 3 | Y | Y (category 3) |
| 101 | *KCNQ4* | 603537 | AD | Y (600101) | Y | Y | Evidential level 3 | Y | Y (category 2) |
| 102 | *KIT* | 164920 | AR | | | | | | |
| 103 | *KITLG* | 184745 | AD | Y (616697) | Y | Y | | | Y (category 2) |
| 104 | *LARS2* | 604544 | AR | Y (615300) | Y | Y | Evidential level 2 | Y | Y (category 1) |
| 105 | *LHFPL5* | 609427 | AR | Y (610265) | Y | Y | Evidential level 3 | Y | Y (category 1) |
| 106 | *LHX3* | 600577 | AR | Y (221750) | | Y | Evidential level 3 | | |
| 107 | *LMX1A* | 600298 | AD | Y (601412) | Y | Y | | | |
| 108 | *LOXHD1* | 613072 | AR | Y (613079) | Y | Y | Evidential level 3 | Y | Y (category 3) |
| 109 | *LRTOMT* | 612414 | AR | Y (611451) | Y | Y | Evidential level 3 | Y | Y (category 3) |
| 110 | *MANBA* | 609489 | AR | Y (248510) | | Y | Evidential level 3 | | |
| 111 | *MARVELD2* | 610572 | AR | Y (610153) | Y | Y | Evidential level 3 | Y | Y (category 2) |
| 112 | *MASP1* | 600521 | AR | Y (257920) | | Y | | | |
| 113 | *MCM2* | 116945 | AD | Y (616968) | Y | Y | | | |
| 114 | *MET* | 164860 | AR | Y (616705) | Y | Y | | | |
| 115 | *MIR96* | 611606 | AD | Y (613074) | Y | Y | Evidential level 3 | | |
| 116 | *MITF* | 156845 | AD/AR | Y (103500, 193510, 617306) | Y | Y | Evidential level 3 | Y | Y (category 3) |
| 117 | *MPZL2* | 604873 | AR | Y (618145) | Y | Y | | | |
| 118 | *MSRB3* | 613719 | AR | Y (613718) | Y | Y | Evidential level 2 | Y | Y (category 3) |
| 119 | *MTAP* | 156540 | AD | | | | Evidential level 1 | | |
| 120 | *MYH14* | 608568 | AD | Y (600652) | Y | Y | Evidential level 2 | Y | Y (category 2) |
| 121 | *MYH9* | 160775 | AD | Y (603622) | Y | Y | Evidential level 3 | Y | Y (category 1) |
| 122 | *MYO15A* | 602666 | AR | Y (600316) | Y | Y | Evidential level 3 | Y | Y (category 1) |
| 123 | *MYO1C* | 606538 | AD | | | | Evidential level 1 | | |
| 124 | *MYO1F* | 601480 | AD | | | | Evidential level 1 | | |
| 125 | *MYO3A* | 606808 | AR | Y (607101) | Y | Y | Evidential level 3 | Y | Y (category 1) |
| 126 | *MYO6* | 600970 | AD/AR | Y (606346, 607821) | Y | Y | Evidential level 3 | Y | Y (category 2) |
| 127 | *MYO7A* | 276903 | AD/AR | Y (601317, 600060) | Y | Y | Evidential level 3 | Y | Y (category 3) |
| 128 | *NARS2* | 612803 | AR | Y (618434, 616239) | Y | Y | | | |
| 129 | *NDP* | 300658 | XR | Y (310600) | Y | Y | Evidential level 3 | | |
| 130 | *NLRP3* | 606416 | AD | Y (617772) | Y | Y | Evidential level 3 | | Y (category 2) |
| 131 | *NR4A2* | 601828 | AD | | | | | | |
| 132 | *OSBPL2* | 606731 | AD | Y (616340) | Y | Y | | Y | Y (category 3) |
| 133 | *OTOA* | 607038 | AR | Y (607039) | Y | Y | Evidential level 3 | Y | Y (category 3) |
| 134 | *OTOF* | 603681 | AR | Y (601071) | Y | Y | Evidential level 3 | Y | Y (category 3) |
| 135 | *OTOG* | 604487 | AR | Y (614945) | Y | Y | Evidential level 2 | Y | Y (category 3) |
| 136 | *OTOGL* | 614925 | AR | Y (614944) | Y | Y | Evidential level 2 | Y | Y (category 1) |
| 137 | *P2RX2* | 600844 | AD | Y (608224) | Y | Y | Evidential level 3 | Y | Y (category 3) |
| 138 | *PAX3* | 606597 | AD | Y (122880, 193500) | Y | Y | Evidential level 3 | Y | Y (category 3) |
| 139 | *PCDH15* | 605514 | AR | Y (609533) | Y | Y | Evidential level 3 | Y | Y (category 3) |
| 140 | *PDE1C* | 602987 | AD | Y (618140) | Y | Y | | | |
| 141 | *PDZD7* | 612971 | AR | Y (618003) | Y | Y | Evidential level 1 | Y | Y (category 3) |
| 142 | *PITX2* | 601542 | AD | | | | | | |
| 143 | *PMP22* | 601097 | AD/AR | Y (145900, 118300) | | | Evidential level 3 | | |
| 144 | *PNPT1* | 610316 | AR | Y (614934) | Y | Y | Evidential level 1 | | |
| 145 | *POLR1C* | 610060 | AR | Y (248390) | Y | Y | Evidential level 2 | | |
| 146 | *POLR1D* | 613715 | AD/AR | Y (613717) | Y | Y | Evidential level 3 | | |
| 147 | *POU3F4* | 300039 | XR | Y (304400) | Y | Y | Evidential level 3 | Y | Y (category 1) |
| 148 | *POU4F3* | 602460 | AD | Y (602459) | Y | Y | Evidential level 3 | Y | Y (category 1) |
| 149 | *PPIP5K2* | 611648 | AR | Y (618422) | Y | Y | | | |
| 150 | *PRPS1* | 311850 | XL | Y (304500) | Y | Y | Evidential level 3 | Y | Y (category 2) |
| 151 | *PTPRQ* | 603317 | AR | Y (617663, 613391) | Y | Y | Evidential level 2 | Y | Y (category 1) |
| 152 | *RDX* | 179410 | AR | Y (611022) | Y | Y | Evidential level 2 | Y | Y (category 3) |
| 153 | *REST* | 600571 | AD | Y (612431) | Y | Y | | | |
| 154 | *ROR1* | 602336 | AR | Y (617654) | Y | Y | | | |
| 155 | *RPGR* | 312610 | XR | Y (300455) | | | | | |
| 156 | *RPS6KA3* | 300075 | XD | Y (303600) | | | | | |
| 157 | *S1PR2* | 605111 | AR | Y (610419) | Y | Y | | Y | Y (category 1) |
| 158 | *SALL1* | 602218 | AD | Y (107480) | | | | | |
| 159 | *SALL4* | 607343 | AD | Y (147750, 607323) | | | | | |
| 160 | *SEMA3E* | 608166 | AD | Y (214800) | | Y | Evidential level 1 | | |
| 161 | *SERPINB6* | 173321 | AR | Y (613453) | Y | Y | Evidential level 2 | Y | Y (category 3) |
| 162 | *SIX1* | 601205 | AD | Y (605192) | Y | Y | Evidential level 3 | Y | Y (category 1) |
| 163 | *SIX5* | 600963 | AD | Y (610896) | Y | Y | Evidential level 1 | | |
| 164 | *SLC12A1* | 600839 | AR | Y (601678) | | | | | |
| 165 | *SLC17A8* | 607557 | AD | Y (605583) | Y | Y | Evidential level 1 | Y | |
| 166 | *SLC19A2* | 603941 | AR | Y (249270) | | Y | Evidential level 3 | | |
| 167 | *SLC22A4* | 604190 | AR | | Y | Y | | | |
| 168 | *SLC26A4* | 605646 | AR | Y (600791) | Y | Y | Evidential level 3 | Y | Y (category 1) |

| | | | | | | | | | |
|---|---|---|---|---|---|---|---|---|---|
| 169 | *SLC26A5* | 604943 | AR | Y (613865) | Y | Y | Evidential level 1 | | |
| 170 | *SLC29A3* | 612373 | AR | Y (602782) | | | | | |
| 171 | *SLC4A11* | 610206 | AR | Y (217400) | | Y | Evidential level 3 | Y | |
| 172 | *SLC52A2* | 607882 | AR | Y (614707) | | Y | | Y | Y (category 2) |
| 173 | *SLITRK6* | 609681 | AR | Y (221200) | | Y | Evidential level 2 | Y | Y (category 1) |
| 174 | *SMPX* | 300226 | XD | Y (300066) | Y | Y | Evidential level 3 | Y | Y (category 1) |
| 175 | *SNAI2* | 602150 | AR | Y (608890) | Y | Y | Evidential level 1 | Y | Y (category 1) |
| 176 | *SOX10* | 602229 | AD | Y (609136, 611584, 613266) | Y | Y | Evidential level 3 | Y | Y (category 1) |
| 177 | *SPINK5* | 605010 | AR | | | | Evidential level 3 | | |
| 178 | *SPNS2* | 612584 | AR | Y (618457) | Y | Y | | | |
| 179 | *STRC* | 606440 | AR | Y (603720) | Y | Y | Evidential level 3 | Y | Y (category 1) |
| 180 | *SYNE4* | 615535 | AR | Y (615540) | Y | Y | Evidential level 2 | Y | Y (category 2) |
| 181 | *TBC1D24* | 613577 | AD/AR | Y (614617, 616044) | Y | Y | Evidential level 3 | Y | Y (category 2) |
| 182 | *TBL1X* | 300196 | XL | Y (301033) | | Y | Evidential level 1 | | |
| 183 | *TCF21* | 603306 | AD | | | | Evidential level 1 | | |
| 184 | *TCOF1* | 606847 | AD | Y (154500) | Y | Y | Evidential level 3 | | |
| 185 | *TECTA* | 602574 | AD/AR | Y (601543, 603629) | Y | Y | Evidential level 3 | Y | Y (category 1) |
| 186 | *TFAP2A* | 107580 | AD | Y (113620) | | Y | Evidential level 2 | | |
| 187 | *TIMM8A* | 300356 | XR | Y (304700) | | Y | Evidential level 3 | Y | Y (category 3) |
| 188 | *TJP2* | 607709 | AR | | Y | Y | Evidential level 2 | | |
| 189 | *TMC1* | 606706 | AD/AR | Y (606705, 600974) | Y | Y | Evidential level 3 | Y | Y (category 1) |
| 190 | *TMC2* | 606707 | AD/AR | | | | | | |
| 191 | *TMEM132E* | 616178 | AR | Y (618481) | Y | Y | | | |
| 192 | *TMIE* | 607237 | AR | Y (600971) | Y | Y | Evidential level 3 | Y | Y (category 1) |
| 193 | *TMPRSS3* | 605511 | AR | Y (601072) | Y | Y | Evidential level 3 | Y | Y (category 3) |
| 194 | *TMPRSS5* | 606751 | AR | | | | | | |
| 195 | *TNC* | 187380 | AD | Y (615629) | Y | Y | Evidential level 1 | | |
| 196 | *TPRN* | 613354 | AR | Y (613307) | Y | Y | Evidential level 3 | Y | Y (category 1) |
| 197 | *TRIOBP* | 609761 | AR | Y (609823) | Y | Y | Evidential level 3 | Y | Y (category 3) |
| 198 | *TRMU* | 610230 | M | Y (580000) | | | | | |
| 199 | *TSPEAR* | 612920 | AR | Y (614861) | Y | Y | Evidential level 2 | Y | |
| 200 | *TUBB4B* | 602660 | AD | Y (617879) | | Y | | | |
| 201 | *TYR* | 606933 | AR | Y (606952) | | | | | |
| 202 | *USH1C* | 605242 | AR | Y (602092) | Y | Y | Evidential level 3 | Y | Y (category 3) |
| 203 | *USH1G* | 607696 | AR | Y (606943) | Y | Y | Evidential level 3 | Y | Y (category 2) |
| 204 | *USH2A* | 608400 | AR | Y (276901) | Y | Y | Evidential level 3 | Y | Y (category 2) |
| 205 | *WBP2* | 606962 | AR | Y (617639) | Y | Y | | | |
| 206 | *WFS1* | 606201 | AD | Y (600965) | Y | Y | Evidential level 3 | Y | Y (category 2) |
| 207 | *WHRN* | 607928 | AR | Y (607084) | Y | Y | Evidential level 3 | Y | Y (category 3) |

Abbreviations: AD, autosomal dominnat; AR, autosomal recessive; XR, X-recessive; XL, X-linked; M, mitochondrial; OMIM, Online Mendelian Inheritance in Man;

HHL, Hereditary Hearing Loss database; DVD, Deafness Variation Database; Y, yes.

References: #1, Abou Tayoun et al. Genet Med. 2016 Jun;18(6):545-53.; #2, Guan et al. Genet Med. 2018 Mar 29. doi: 10.1038/gim.2018.48.; #3, DiStefano et al. J Mol Diagn. 2018 Nov;20(6):789-801.

**Supplementary Table S2. Quality control metrics of NGS test results in this study.**

| Test modality | Parameters | Total reads | Average depth | % Covered (>20×) | % Covered (>30×) |
|---|---|---|---|---|---|
| Panel | Mean | 12,542,376 bp | 1335.3 x | 100.0% | 99.7% |
| | Standard deviation | 556,803 bp | 49.6 x | 0.0% | 0.1% |
| Exome | Mean | 72,127,477 bp | 83.5 x | 98.4% | 95.8% |
| | Standard deviation | 8,347,293 bp | 34.5 x | 0.1% | 0.3% |

**Supplementary Table S3. Primers for real-time PCR performed in this study.**

| Gene | Species | Forward sequence | Reverse sequence |
|---|---|---|---|
| *HES1* | Human | GGAAATGACAGTGAAGCACCTCC | GAAGCGGGTCACCTCGTTCATG |
| *HES5* | Human | TCCTGGAGATGGCTGTCAGCTA | CGTGGAGCGTCAGGAACTGCA |
| *Dll1* | Mouse | GCTGGAAGTAGATGAGTGTGCTC | CACAGACCTTGCCATAGAAGCC |
| *Notch1* | Mouse | GCTGCCTCTTTGATGGCTTCGA | CACATTCGGCACTGTTACAGCC |
| *Hes1* | Mouse | GGAAATGACTGTGAAGCACCTCC | GAAGCGGGTCACCTCGTTCATG |

**Supplementary Table S4. Antibodies and peptides used in this study.**

| Target protein | Catalog number | Manufacturer | Dilution (Use) |
|---|---|---|---|
| anti-DLL1 | ab10554, ab85346 | Abcam | 1:1000 (WB), 1:200 (IF) |
| anti-HES1 | ab71559 | Abcam | 1:1000 (WB) |
| anti-HES5 | ab25374 | Abcam | 1:1000 (WB) |
| anti-cleaved Notch1 (anti-NICD) | 4147 | Cell Signaling Technology | 1:1000 (WB) |
| anti-Notch1 | sc-6014 | Santa Cruz | 1:1000 (WB), 1:200 (IF) |
| anti-aldolase A1 | sc-12059 | Santa Cruz | 1:1000 (WB) |
| anti-GM130 | 610823 | BD Biosciences | 1:200 (IF) |

| Treated peptide | Sequence information |
|---|---|
| DLL1 peptide | SGVFELKLQEFVNKKGLLGNRNCCRGGAGPPPCACRTFFRVCLKHYQASVSPEPPCT<br>YGSAVTPVLGVDSFSLPDGGGADSAFSNPIRFPFGFTWPGTFSLIIEALHTDSPDDLAT<br>ENPERLISRLATQRHLTVGEEWSQDLHSSGRTDLKYSYRFVCDEHYYGEGCSVFCRPR<br>DDAFGHFTCGERGEKVCNPGWKGPYCTEPICLPGCDEQHGFCDKPGECKCRVGWQ<br>GRYCDECIRYPGCLHGTCQQPWQCNCQEGWGGLFCNQDLNYCTHHKPCKNGAT<br>CTNTGQGSYTCSCRPGYTGATCELGIDECDPSPCKNGGSCTDLENSYSCTCPPGFYG<br>KICELSAMTCADGPCFNGGRCSDSPDGGYSCRCPVGYSGFNCEKKIDYCSSSPCSNG<br>AKCVDLGDAYLCRCQAGFSGRHCDDNVDDCASSPCANGGTCRDGVNDFSCTCPP<br>GYTGRNCSAPVSRCEHAPCHNGATCHERGHRYVCECARGYGGPNCQFLLPELPPGP<br>AVVDLTEKLEGQGGPF |
| control peptide | MAEAPPRRLGLGPPPGDAPRAELVALTAVQSEQGEAGGGGSPRRLGPLGSPLPPGAP<br>LPGPGSGSGSACGQRSSAAHKRYRRLQNWVYNVLER |

**Supplementary Table S5. Profiles of clinical characteristics and pathogenic/likely pathogenic mutations identified in patients with post-lingual hearing loss.**

| Gene | YUHL patient number | Age of onset | Audiogram pattern group | Platform | Mode of inheritance | NM number | hg19 position | Nucleotide change | Amino acid change | Zygosity | HL-specified ACMG classification | HL-specified ACMG components | gnomAD MAF | gnomAD maximum MAF in subpopulation | In silico | REVEL score | Number of segregation | Novel (reported PMID) |
|---|---|---|---|---|---|---|---|---|---|---|---|---|---|---|---|---|---|---|
| **Inheritance mode: Autosomal dominant genes** | | | | | | | | | | | | | | | | | | |
| *ACTG1* | YUHL255 | late 10s | ski-slope | ES | AD | NM_001199954.1 | chr17:79479168C>T | c.124G>A | p.G42S | Het | Likely pathogenic | PM2, PP1_S, PP3 | 0 | NA | T/D/D /*** | 0.768 | 5 | Yes |
| *ACTG1* | YUHL153 | early 40s | other | ES | AD | NM_001199954.1 | chr17:79477831G>A | c.1013C>T | p.S338L | Het | Likely pathogenic | PM2, PP1_S, PP3 | 0 | NA | D/D/D | 0.883 | 6 | Yes |
| *COCH* | YUHL195 | late 30s | other | ES | AD | NM_004086.2 | chr14:31346808G>A | c.113G>A | p.G38D | Het | Likely pathogenic | PS1, PS3_M, PM2, PP1_M, PP3 | 0 | NA | T/D/D | 0.721 | 4 | No (23990876, 26758463) |
| *COCH* | YUHL104 | late 40s | other | ES | AD | NM_004086.2 | chr14:31346921G>A | c.226G>A | p.A76T | Het | Likely pathogenic | PS1, PS3_M, PM2, PP3 | 0 | NA | T/D/D | 0.802 | 1 | No (26969326) |
| *COCH* | YUHL5* | late 40s | ski-slope | ES | AD | NM_004086.2 | chr14:31348145T>A | c.368T>A | p.V123E | Het | Likely pathogenic | PS1, PS3_M, PM2, PP1_S | 0 | NA | D/B/D | 0.625 | 7 | No (26256111) |
| *COCH* | YUHL590 | early 60s | ski-slope | Panel | AD | NM_004086.2 | chr14:31353819T>G | c.690T>G | p.F230L | Het | Likely pathogenic | PS1, PS3_M, PM2, PP1_S | 0 | NA | T/D/D | 0.256 | 5 | Yes |
| *COCH* | YUHL102 | early 30s | other | ES | AD | NM_004086.2 | chr14:31355248G>T | c.1207G>T | p.G403C | Het | Likely pathogenic | PS3_M, PM2, PP1_S | 0 | NA | D/D/D | 0.619 | 10 | Yes |
| *COCH* | YUHL612 | early 40s | other | Panel | AD | NM_004086.2 | chr14:31355381G>A | c.1340G>A | p.G447D | Het | Likely pathogenic | PS3_M, PM2, PP1_M, PP3 | 0 | NA | T/D/D | 0.934 | 4 | Yes |
| *COCH* | YUHL265 | late 50s | ski-slope | ES | AD | NM_004086.2 | chr14:31358924T>G | c.1580T>G | p.F527C | Het | Pathogenic | PS1, PS3_M, PS4_P, PM2, PP1_M, PP3 | 0 | NA | D/D/D | 0.955 | 4 | No (22610276) |
| *COCH* | YUHL522 | late 10s | ski-slope | ES | AD | NM_004086.2 | chr14:31358924T>G | c.1580T>G | p.F527C | Het | Pathogenic | PS1, PS3_M, PS4_P, PM2, PP1_M, PP3 | 0 | NA | D/D/D | 0.955 | 4 | No (22610276) |
| *COL11A2* | YUHL161* | early 50s | ski-slope | Panel | AD | NM_080680.2 | chr6:33139557G>A | c.3083C>T | p.P1028L | Het | Likely pathogenic | PS4_P, PM2, PP3 | 0.00041% (het only) | 0.00005477 | D/D/D | 0.794 | 0 | No (31045651) |
| *COL1A1* | YUHL61 | late 10s | other | ES | AD | NM_000088.3 | chr17:48265907del | c.3191delG | p.G1064Vfs*44 | Het | Pathogenic | PVS1, PM2, PP1 | 0 | NA | NA | NA | 2 | Yes |
| *COL4A3* | YUHL408 | early 50s | other | Panel | AD | NM_000091.3 | chr2:228159760G>A | c.3499G>A | p.G1167R | Het | Likely pathogenic | PS1, PM1, PM2, PP3 | 0 | NA | D/D/D | 0.994 | 0 | No (11134255, 27281700) |
| *COL4A3* | YUHL325 | late 40s | other | ES | AD | NM_000091.3 | chr2:228175529T>G | c.4793T>G | p.L1598R | Het | Likely pathogenic | PS1, PM2, PP1, PP3 | 0 | NA | D/D/D | 0.976 | 2 | No (24633401) |
| *DIAPH1* | YUHL473 | late 50s | other | Panel | AD | NM_005219.4 | chr5:140905652C>T | c.3527G>A | p.R1176Q | Het | Likely pathogenic | PM2, PP1_S | 0.00040% (het only) | 0.000008831 | T/D/D | 0.28 | 6 | Yes |
| *EYA4* | YUHL601 | mid 20s | other | Panel | AD | NM_004100.5 | chr6:133789762C>A | c.863C>A | p.S288* | Het | Pathogenic | PVS1, PS1, PM2, PP1 | 0 | NA | NA | NA | 2 | No (22938506) |
| *EYA4* | YUHL555 | early 50s | other | ES | AD | NM_004100.5 | chr6:133827286A>C | c.1234A>C | p.M412L | Het | Likely pathogenic | PM2, PP1_S | 0 | NA | D/B/D | 0.512 | 5 | Yes |
| *GRHL2* | YUHL400 | late 40s | other | ES | AD | NM_024915.3 | chr8:102643938_102643942del | c.1331_1335delCTCAA | p.T444Mfs*6 | Het | Likely pathogenic | PVS1_S, PM2 | 0 | NA | NA | NA | 0 | Yes |
| *KCNQ4* | YUHL103* | late 50s | ski-slope | ES | AD | NM_004700.3 | chr1:41249905T>C | c.140T>C | p.L47P | Het | Pathogenic | PS1, PS3, PM2, PP1_M | 0 | NA | D/B/D | 0.365 | 4 | No (30556268) |
| *KCNQ4* | YUHL168 | early 10s | other | ES | AD | NM_004700.3 | chr1:41249905T>C | c.140T>C | p.L47P | Het | Pathogenic | PS1, PS3, PM2, PP1_M | 0 | NA | D/B/D | 0.365 | 4 | No (30556268) |
| *KCNQ4* | YUHL192 | early 10s | other | Panel | AD | NM_004700.3 | chr1:41249905T>C | c.140T>C | p.L47P | Het | Pathogenic | PS1, PS3, PM2, PP1 | 0 | NA | D/B/D | 0.365 | 3 | No (30556268) |
| *KCNQ4* | YUHL343 | late 10s | other | ES | AD | NM_004700.3 | chr1:41249905T>C | c.140T>C | p.L47P | Het | Pathogenic | PS1, PS3, PM2, PP1_M | 0 | NA | D/B/D | 0.365 | 4 | No (30556268) |
| *KCNQ4* | YUHL556 | early 50s | ski-slope | ES | AD | NM_004700.3 | chr1:41249905T>C | c.140T>C | p.L47P | Het | Pathogenic | PS1, PS3, PM2, PP1_M | 0 | NA | D/B/D | 0.365 | 4 | No (30556268) |
| *KCNQ4* | YUHL35* | early 30s | other | ES | AD | NM_004700.3 | chr1:41285106G>T | c.796G>T | p.D266Y | Het | Pathogenic | PS1, PS3_M, PM1, PM2, PP1, PP3 | 0 | NA | D/D/D | 0.754 | 2 | No (30413759) |
| *KCNQ4* | YUHL611 | first decade | ski-slope | Panel | AD | NM_004700.3 | chr1:41285632G>T | c.920G>T | p.G307V | Het | Likely pathogenic | PS3_M, PM2, PP1, PP3 | 0 | NA | D/D/D | 0.958 | 3 | Yes |
| *KCNQ4* | YUHL206 | early 50s | other | ES | AD | NM_004700.3 | chr1:41296802C>T | c.1339C>T | p.R447W | Het | Likely pathogenic | PS3_M, PP1_S | 0.0033% (het only) | 0.00007535 | D/B/P | 0.524 | 5 | Yes |
| *MYH14* | YUHL83* | early 60s | ski-slope | ES | AD | NM_001145809.1 | chr19:50766675A>C | c.2692A>C | p.K898Q | Het | Likely pathogenic | PP1_M, PP3, PP4 | 0.002833% (het only) | 0.0003995 | D/D/D | 0.722 | 4 | Yes |
| *MYH14* | YUHL77* | early 30s | ski-slope | ES | AD | NM_001145809.1 | chr19:50774700C>T | c.3068C>T | p.A1023V | Het | Likely pathogenic | PS1, PP1, PP4 | 0.003743% (het only) | 0.0001599 | D/D/D | 0.308 | 2 | No (31045651) |
| *MYH14* | YUHL646 | early 30s | ski-slope | Panel | AD | NM_001145809.1 | chr19:50792796C>G | c.4856C>G | p.A1619G | Het | Likely pathogenic | PM2, PP1_S, PP4 | 0 | NA | D/D/D | 0.446 | 5 | Yes |
| *MYH9* | YUHL91* | early 30s | other | ES | AD | NM_002473.4 | chr22:36689501C>A | c.3969G>T | p.Q1323H | Het | Likely pathogenic | PM2, PP1_S | 0 | NA | D/D/D | 0.497 | 5 | No (31045651) |
| *MYH9* | YUHL410 | late 50s | other | Panel | AD | NM_002473.4 | chr22:36681790G>A | c.5188C>T | p.R1730C | Het | Likely pathogenic | PS1, PS4_P, PP3 | 0.01441% (het only) | 0.001222 | D/D/D | 0.85 | 0 | No (27393652) |
| *MYH9* | YUHL528 | mid 40s | ski-slope | ES | AD | NM_002473.4 | chr22:36681790G>A | c.5188C>T | p.R1730C | Het | Likely pathogenic | PS1, PS4_P, PP3 | 0.01441% (het only) | 0.001222 | D/D/D | 0.85 | 1 | No (27393652) |
| *MYO6* | YUHL633 | early 10s | other | Panel | AD | NM_004999.3 | chr6:76550326del | c.578delG | p.G193Efs*18 | Het | Likely pathogenic | PVS1_M, PS1, PM2, PP1 | 0 | NA | NA | NA | 3 | No (32143290) |
| *MYO7A* | YUHL541 | mid 50s | other | ES | AD | NM_000260.3 | chr11:76883843G>A | c.1847G>A | p.R616Q | Het | Likely pathogenic | PM2, PM5, PP1, PP4 | 0.0051% (het only) | 0.0001163 | T/D/D | 0.291 | 2 | Yes |
| *MYO7A* | YUHL550 | mid 10s | ski-slope | ES | AD | NM_000260.3 | chr11:76901135C>G | c.3701C>G | p.T1234S | Het | Likely pathogenic | PS1, PM2, PP1 | 0.0036% (het only) | 0.0004964 | T/D/D | 0.689 | 2 | No (25373420) |
| *MYO7A* | YUHL308 | early 10s | ski-slope | Panel | AD | NM_000260.3 | chr11:76917162C>T | c.5657C>T | p.P1886L | Het | Likely pathogenic | PS4_P, PM2, PP1_M, PP3 | 0 | NA | D/D/D | 0.795 | 4 | Yes |
| *NLRP3* | YUHL282 | early 20s | other | Panel | AD | NM_004895.4 | chr1:247586640A>G | c.392A>G | p.K131R | Het | Likely pathogenic | PS1, PP1_M | 0.0060% (het only) | 0.0008155 | T/B/P | 0.048 | 4 | No (31155445) |
| *NLRP3* | YUHL185 | early 20s | ski-slope | Panel | AD | NM_004895.4 | chr1:247587682G>A | c.937G>A | p.E313K | Het | Likely pathogenic | PS1, PM2, PP1 | 0 | NA | D/D/D | 0.578 | 3 | No (25596455, 27191192) |
| *NLRP3* | YUHL481 | first decade | other | Panel | AD | NM_004895.4 | chr1:247588381G>A | c.1636G>A | p.V546I | Het | Likely pathogenic | PS3_P, PP1_S, PP4 | 0.0053% (het only) | 0.0001976 | T/B/P | 0.191 | 5 | Yes |
| *NLRP3* | YUHL262 | early 30s | ski-slope | ES | AD | NM_004895.4 | chr1:247597509G>T | c.2432G>T | p.G811V | Het | Likely pathogenic | PS4_M, PM2, PP1_M | 0 | NA | D/B/D | 0.344 | 4 | Yes |
| *OPA1* | YUHL31 | early 30s | other | ES | AD | NM_130837.2 | chr3:193355762A>C | c.1057A>C | p.S353R | Het | Likely pathogenic | PM2, PP1_M, PP3, PP4 | 0 | NA | D/D/D | 0.965 | 4 | Yes |
| *OSBPL2* | YUHL3 | early 10s | ski-slope | ES | AD | NM_144498.2 | chr20:60835157_60835158del | c.158_159delAA | p.Q53Rfs*100 | Het | Pathogenic | PS1, PS3, PM2, PP1_S | 0 | NA | NA | NA | 12 | No (30894143) |
| *OSBPL2* | YUHL457 | early 40s | ski-slope | ES | AD | NM_144498.2 | chr20:60835182_60835183del | c.180_181delCA | p.H60Qfs*93 | Het | Pathogenic | PS3, PM2, PP1_S | 0 | NA | NA | NA | 5 | Yes |
| *POU4F3* | YUHL186 | early 30s | other | Panel | AD | NM_002700.2 | chr5:145719215del | c.225delG | p.K75Nfs*9 | Het | Likely pathogenic | PVS1_M, PM2, PP1_M | 0 | NA | NA | NA | 4 | Yes |
| *POU4F3* | YUHL365 | early 30s | other | ES | AD | NM_002700.2 | chr5:145719459C>T | c.469C>T | p.Q157* | Het | Likely pathogenic | PVS1_M, PM2, PP1_M | 0 | NA | NA | NA | 4 | Yes |
| *TECTA* | YUHL171 | early 50s | other | Panel | AD | NM_005422.2 | chr11:121008574C>G | c.3386C>G | p.T1129R | Het | Likely pathogenic | PM2, PP1_S | 0.00040% (het only) | 0.00005438 | T/D/D | 0.369 | 5 | Yes |
| *TECTA* | YUHL588 | mid 10s | other | Panel | AD | NM_005422.2 | chr11:121038767A>G | c.5591A>G | p.N1864S | Het | Likely pathogenic | PS1, PM2, PP1 | 0 | NA | D/B/D | 0.472 | 2 | No (26969326) |
| *TECTA* | YUHL553 | early 20s | ski-slope | ES | AD | NM_005422.2 | chr11:121058603G>A | c.6062G>A | p.R2021H | Het | Pathogenic | PS1, PS4_M, PM2, PP1, PP3 | 0.00040% (het only) | 0.000008791 | T/D/D | 0.825 | 3 | No (12162770) |
| *TECTA* | YUHL194 | late 20s | other | ES | AD | NM_005422.2 | chr11:120984347C>T | c.710C>T | p.T237I | Het | Likely pathogenic | PS1, PM2, PP1, PP3 | 0 | NA | D/D/D | 0.901 | 3 | No (24816743) |
| *TECTA* | YUHL517 | early 10s | ski-slope | Panel | AD | NM_005422.2 | chr11:120984429T>G | c.790+2T>G | splice | Het | Likely pathogenic | PM2, PP1_S | 0 | NA | NA | NA | 5 | Yes |
| *WFS1* | YUHL208 | late 20s | other | ES | AD | NM_006005.3 | chr4:6302546G>A | c.1024G>A | p.A342T | Het | Likely pathogenic | PP1_S, PP1, PP4 | 0.025% (het only) | 0.0006859 | D/D/D | 0.562 | 7 | Yes |
| *WFS1* | YUHL430 | mid 10s | other | Panel | AD | NM_006005.3 | chr4:6302991C>A | c.1469C>A | p.T490N | Het | Likely pathogenic | PM2, PP1_M, PS4_P, PP4 | 0 | NA | D/B/D | 0.421 | 4 | Yes |
| *WFS1* | YUHL30 | first decade | other | ES | AD | NM_006005.3 | chr4:6303941A>C | c.2419A>C | p.S807R | Het | Likely pathogenic | PS1, PM2, PP1_M, PP3, PP4 | 0 | NA | T/D/D | 0.875 | 4 | No (12073007, 29258540) |
| **Inheritance mode: Autosomal recessive genes** | | | | | | | | | | | | | | | | | | |
| *ADGRV1* | YUHL422 | late 40s | ski-slope | ES | AR | NM_032119.3 | chr5:89943537C>T | c.3245C>T | p.P1082L | Het | Likely pathogenic | PM2, PM3, PP1_M | 0.0020% (het only) | 0.00005794 | D/D/D | 0.44 | 2 | Yes |
| | | | | | | | chr5:89985711T>C | c.6524T>C | p.V2175A | Het | Likely pathogenic | PM2, PM3, PP1_M | 0.0016% (het only) | 0.0002231 | T/D/D | 0.32 | 2 | Yes |
| *GJB2* | YUHL510 | late 10s | ski-slope | Panel | AR | NM_004004.5 | chr13:20763612C>T | c.109G>A | p.V37I | Het | Pathogenic | PS3_M, PS4, PM3_VS, PM5, PP1_S | 0.7556% | 0.08345 | D/D/D | 0.656 | 3 | No (31160754) |
| | | | | | | | chr13:20763294G>A | c.427C>T | p.R143W | Het | Pathogenic | PS3_M, PS4, PM3_VS, PP1_S, PP3 | 0.01311% (het only) | 0.0007227 | D/D/D | 0.918 | 3 | No (32120898) |
| *MYO15A* | YUHL339 | mid 40s | other | ES | AR | NM_016239.3 | chr17:18023299dup | c.1185dupC | p.E396Rfs*36 | Het | Pathogenic | PVS1, PS1, PM2, PM3, PP1_M | 0.0061% (het only) | 0.0003075 | NA | NA | 2 | No (22245518) |
| | | | | | | | chr17:18042917C>T | c.5203C>T | p.R1735W | Het | Likely pathogenic | PS1, PM2, PM3, PP3 | 0.0014% (het only) | 0.00006544 | D/D/D | 0.762 | 1 | No (28964305) |
| *MYO15A* | YUHL604 | early 10s | ski-slope | Panel | AR | NM_016239.3 | chr17:18054742C>T | c.7688C>T | p.T2563M | Het | Likely pathogenic | PM2, PM3_S | 0.0012% (het only) | 0.0001112 | D/B/P | 0.211 | 0 | Yes |
| | | | | | | | chr17:18075517C>G | c.10263C>G | p.I3421M | Het | Likely pathogenic | PS1, PM2 | 0.0028% (het only) | 0.0003894 | D/D/D | 0.582 | 0 | No (29482514) |
| *OTOA* | YUHL293 | first decade | ski-slope | ES | AR | NM_144672.3 | chr16:21730784del | c.1765delC | p.Q589Rfs*55 | Het | Pathogenic | PVS1, PS1, PM2, PP1 | 0.0012% (het only) | 0.0001631 | NA | NA | 1 | No (25373420, 30740825) |
| | | | | | | | chr16:21742199del | c.2249delG | p.G750Dfs*4 | Het | Pathogenic | PVS1, PM2, PM3, PP1 | 0 | NA | NA | NA | 1 | Yes |
| *OTOA* | YUHL524 | early 10s | other | ES | AR | NM_144672.3 | chr16:21721369G>T | c.1265G>T | p.G422V | Hemi | Likely pathogenic | PM2, PM3, PP1, PP4 | 0.0050% (het only) | 0.0006531 | D/D/D | 0.48 | 1 | Yes |
| | | | | | | | NA | whole gene deletion | | Het | Pathogenic** | score=1.45 (1A, 2A, 3A, 4L, 5H) | 0.08758% (het only) | NA | NA | NA | 2 | No (31527525) |
| *OTOGL* | YUHL418 | early 10s | other | Panel | AR | NM_173591.3 | chr12:80752491_80752492del | c.6135_6136delTG | p.A2046Rfs*21 | Homo | Pathogenic | PVS1, PM2, PM3_P, PP1 | 0.0016% (het only) | 0.0002180 | NA | NA | 1 | Yes |

| | | | | | | | | | | | | | | | | | | |
|---|---|---|---|---|---|---|---|---|---|---|---|---|---|---|---|---|---|---|
| *STRC/ CATSPER2* | YUHL204 | early 10s | ski-slope | Panel | AR | NM_153700.2 | NA | whole gene deletion | | Homo | Pathogenic** | score=1.45 (1A, 2A, 3A, 4L, 5H) | 0.903% (het only) | NA | NA | NA | 0 | No (32203226) |
| *STRC/ CATSPER2* | YUHL695 | first decade | ski-slope | Panel | AR | NM_153700.2 | NA | whole gene deletion | | Homo | Pathogenic** | score=1.45 (1A, 2A, 3A, 4L, 5H) | 0.903% (het only) | NA | NA | NA | 1 | No (32203226) |
| *TMPRSS3* | YUHL154 | late 20s | ski-slope | ES | AR | NM_024022.2 | chr21:43808633G>A | c.325C>T | p.R109W | Het | Likely pathogenic | PS1, PM2_P, PM3, PP3 | 0.01203% (het only) | 0.002025 | D/D/D | 0.767 | 0 | No (12920079, 28566687) |
| | | | | | | | chr21:43808632C>T | c.326G>A | p.R109Q | Het | Likely pathogenic | PS1, PM2_P, PM3 | 0.01167% (het only) | 0.0003668 | D/D/D | 0.563 | 0 | No (24853665) |
| *TMPRSS3* | YUHL630 | early 20s | ski-slope | ES | AR | NM_024022.2 | chr21:43808567C>T | c.391G>A | p.D131N | Het | Likely pathogenic | PM2_P, PM3, PP1_M | 0.01626% (het only) | 0.0004379 | D/D/D | 0.339 | 2 | Yes |
| | | | | | | | chr21:43803196C>T | c.728G>A | p.G243E | Het | Likely pathogenic | PM2, PM5, PP1, PP3 | 0 | NA | D/D/D | 0.968 | 1 | Yes |
| *TMPRSS3* | YUHL240* | first decade | ski-slope | Panel | AR | NM_024022.2 | chr21:43802210C>T | c.916G>A | p.A306T | Het | Likely pathogenic | PS1, PM2_P, PM3, PP1, PP3 | 0.01533% (het only) | 0.0006014 | D/D/D | 0.851 | 1 | No (31045651, 28695016) |
| | | | | | | | chr21:43800235C>A | c.1039G>T | p.E347* | Het | Pathogenic | PVS1, PM2, PM3, PP3 | 0 | NA | D/D/D | NA | 0 | No (31045651) |
| *USH2A* | YUHL116* | early 10s | ski-slope | Panel | AR | NM_206933.2 | chr1:216424330A>C | c.2802T>G | p.C934W | Het | Pathogenic | PS1, PM2, PM3_P, PP1, PP3, PP4 | 0 | NA | D/D/D | 0.817 | 1 | No (31045651, 31456290) |
| | | | | | | | chr1:215931934T>A | c.11389+3A>T | splice | Het | Pathogenic | PS1, PM2, PM3_P, PM5, PP4 | 0.00080% (het only) | 0.00005442 | NA | NA | 0 | No (31045651, 32093671) |
| *USH2A* | YUHL228 | late 20s | other | Panel | AR | NM_206933.2 | chr1:215821895T>C | c.14557A>G | p.M4853V | Het | Likely pathogenic | PS1, PM2, PM3_P, PP4 | 0.0052% (het only) | 0.0006525 | T/B/P | 0.029 | 0 | No (29178603) |
| | | | | | | | chr1:216017635C>A | c.9258+1G>T | splice | Het | Likely pathogenic | PS1, PM2, PM5, PP4 | 0 | NA | NA | NA | 0 | No (30902645) |
| **Inheritance mode: X-linked dominant genes** | | | | | | | | | | | | | | | | | | |
| *COL4A6* | YUHL455 | first decade | other | ES | XD | NM_001287758.1 | chrX:107454918C>T | c.494G>A | p.S165N | Het | Likely pathogenic | PS3_P, PP1_S, PP4 | 0.013% (het only) | 0.001663 | T/D/P | 0.116 | 5 | Yes |

NCBI gene reference sequences (NM) is provided. gnomAD database of v2.1.1 and SVs v2.1 were used. Hearing loss-specified ACMG classification was comprehensively applied according to ref 8. In silico prediction results of SIFT, PolyPhen-2, and Mutation Taster are provided.

*Previously reported patients in the publications of ref 15 and two reports (PMID: 30413759, 30556268).

** ACMG guideline for copy number variation (PMID: 31690835) was applied for the variants, all of which were confirmed by multiplex ligation-dependent probe amplification.

*** Five splice site prediction tools provided no significantly differential values suggesting unlikeliness of alternative splicing due to the missense variant (NNSPLICE: 0.2, MaxEntScan: 0.5, GeneSplicer: 0.6, Human Splicing Finder: 3.2, SpliceSiteFinder-like: 3.9).

Abbreviations: ACMG, American College of Medical Genetics and Genomics; AD, autosomal dominant; AR, autosomal recessive; B, benign; D, deleterious or damaging; ES, exome sequencing; Hemi, hemizygous; Het, heterozygous; Hom, homozygous; MAF, minor allele frequency; NA, not applicable; T, tolerated; XD, X-linked dominant; YUHL, Yonsei University Hearing Loss cohort.

**Supplementary Table S6. Summary of top five candidate genes from the candidate gene prioritization process using gene-by-gene analysis, variant-level analysis, and patient-based analysis.**

| Gene | Gene-by-gene analysis | | | | Variant-level analysis | | | Patient-based analysis | |
|---|---|---|---|---|---|---|---|---|---|
| | OMIM database gene-disease association, inheritance mode | IMPC, MGI mouse model assessed for hearing function | gnomAD total number of loss of function variants | PubMed publication associated with gene and hearing | Variant types | Conservation status | genomAD allele frequency for variants | Autosomal dominant inheritance pattern | Progressive nature of hearing loss |
| *DLL1* | Neurodevelopmental disorder, AD (registered in Dec 2019) | Yes (Knock-out; abnormal hearing) | 0 | PMID: 28676722, 27168786 | Missense, Missense | Danio rerio | 0.0028%, 0.0029% | Yes | Yes |
| *NFKBIZ* | No associated disease | No | 8 | x | Missense, Missense | Xenopus tropicalis | 0, 0.00081% | No | Yes |
| *ASTN1* | No associated disease | No | 9 | x | Missense, Missense | Danio rerio | 0.0077%, 0.00081% | Yes | Yes |
| *ARHGAP1* | No associated disease | No | 10 | x | Missense, Missense | Danio rerio | 0.0086%, 0.0014% | No | Yes |
| *TTC28* | No associated disease | No | 21 | PMID: 22436304 | Missense, Missense | Danio rerio | 0.016%, 0 | Yes | Yes |

**Supplementary Table S7. Heterozygous variants in *DLL1* detected in three patients with ski-slope hearing loss.**

| Individual | Sex | Age of onset (years) | Nucleotide change[a] | Amino acid change | Exon[a] | Zygosity | dbSNP[b] | ESP[c] | gnomAD[d] | KRGDB[e] | PP2[f] | MT[g] | Condel[h] | SIFT[i] | CADD[j] | HGMD[k] | ClinVar[l] |
|---|---|---|---|---|---|---|---|---|---|---|---|---|---|---|---|---|---|
| YUHL106 II-1 | M | Mid 20s | c.1195G>A | p.Gly399Ser | 8/11 | Het | rs112145095 | ND | 0.00000028 | 0.000588 | Dam | DC | Del | Del | 28.1 | ND | ND |
| YUHL196<br>I-2<br>II-1<br>II-2 | <br>F<br>F<br>F | <br>Early 40s<br>Early 40s<br>Early 30s | c.1930G>A | p.Val644Met | 9/11 | <br>Het<br>Het<br>Het | rs187849210 | ND | 0.000029 | 0.000291 | Dam | DC | Del | Del | 23.1 | ND | ND |
| YUHL80 II-1 | M | Early 40s | c.1409C>T | p.Pro470Leu | 9/11 | Het | rs200081575 | ND | 0.0003628 | 0.004368 | Dam | DC | Del | Tol | 24.4 | ND | ND |

Dam, probably damaging; Del, deleterious; DC, disease-causing; Het, heterozygous in the affected individual; F, female; M, male; ND, no data.

[a]cDNA variants are numbered according to human cDNA reference sequence NM_005618.4 (DLL1); +1 corresponds to the A of the ATG translation initiation codon.

[b]dbSNP database (http://www.ncbi.nlm.nih.gov/SNP).

[c]NHLBI Exome Sequencing Project (http://evs.gs.washington.edu/EVS/).

[d]gnomAD browser (http://exac.broadinstitute.org/).

[e]The Korean Reference Genome Database.

[f]PolyPhen-2 HumVar prediction score (http://genetics.bwh.harvard.edu/pph2/).

[g]Mutation taster (http://www.mutationtaster.org/).

[h]Condel (http://bbglab.irbbarcelona.org/fannsdb/).

[i]SIFT, Sorting Intolerant from Tolerant (http://sift.jcvi.org/).

[j]CADD, Phred-like scores (scaled C scores) on Combined Annotation Dependent Depletion (http://cadd.gs.washington.edu/home/).

[k]HGMD, The Human Gene Mutation Database (http://www.hgmd.cf.ac.uk/ac/index.php).

[l]ClinVar (https://www.ncbi.nlm.nih.gov/clinvar/).
